# Exploring IRGs as a Biomarker of Pulmonary Hypertension Using Multiple Machine Learning Algorithms

**DOI:** 10.3390/diagnostics14212398

**Published:** 2024-10-28

**Authors:** Jiashu Yang, Siyu Chen, Ke Chen, Junyi Wu, Hui Yuan

**Affiliations:** Department of Clinical Laboratory Center, Beijing Anzhen Hospital, Capital Medical University, Beijing 100029, China; yangjs2022@mail.ccmu.edu.cn (J.Y.); chens2001@163.com (S.C.); ckdexxy@163.com (K.C.); wujunyi1688@mail.ccmu.edu.cn (J.W.)

**Keywords:** pulmonary arterial hypertension, biomarkers, machine learning, diagnosis, bioinformatics

## Abstract

Background: Pulmonary arterial hypertension (PAH) is a severe disease with poor prognosis and high mortality, lacking simple and sensitive diagnostic biomarkers in clinical practice. This study aims to identify novel diagnostic biomarkers for PAH using genomics research. Methods: We conducted a comprehensive analysis of a large transcriptome dataset, including PAH and inflammatory response genes (IRGs), integrated with 113 machine learning models to assess diagnostic potential. We developed a clinical diagnostic model based on hub genes, evaluating their effectiveness through calibration curves, clinical decision curves, and ROC curves. An animal model of PAH was also established to validate hub gene expression patterns. Results: Among the 113 machine learning algorithms, the Lasso + LDA model achieved the highest AUC of 0.741. Differential expression profiles of hub genes CTGF, DDR2, FGFR2, MYH10, and YAP1 were observed between the PAH and normal control groups. A diagnostic model utilizing these hub genes was developed, showing high accuracy with an AUC of 0.87. MYH10 demonstrated the most favorable diagnostic performance with an AUC of 0.8. Animal experiments confirmed the differential expression of CTGF, DDR2, FGFR2, MYH10, and YAP1 between the PAH and control groups (*p* < 0.05); Conclusions: We successfully established a diagnostic model for PAH using IRGs, demonstrating excellent diagnostic performance. CTGF, DDR2, FGFR2, MYH10, and YAP1 may serve as novel molecular diagnostic markers for PAH.

## 1. Introduction

Pulmonary hypertension (PH) refers to a group of diseases where the mean pulmonary artery pressure (mPAP) exceeds 20 mmHg during periods of rest [1]. One study suggests that pulmonary hypertension (PH) affects at least 1% of the global population [2]. The 2022 ESC/ERS guidelines classify pulmonary hypertension (PH) into five distinct groups, taking into consideration its pathomechanism, clinical manifestations, hemodynamic characteristics, and therapeutic approaches. These groups are pulmonary arterial hypertension (PAH), PH associated with left heart disease, PH associated with lung diseases and/or hypoxia, PH associated with pulmonary artery obstructions, and PH with unclear and/or multifactorial mechanisms [1]. PAH is classified as the first subtype of PH, encompassing various diseases, with idiopathic pulmonary arterial hypertension (IPAH) being the most prevalent. The onset of PAH is gradual and lacks specificity during the early stages, typically presenting as fatigue and shortness of breath [3]. If PAH continues to progress, it can lead to heart failure, posing a significant health risk. The diagnosis of PAH is confirmed through Right Heart Catheterization, a test commonly used for this purpose. However, due to its invasive nature and potential serious complications, this test is typically performed after PAH has already been recognized. As an initial screening tool for PAH, cardiac echocardiography is often utilized. It has a sensitivity of 85% and a specificity of 70–74% in diagnosing PAH [4,5]. It is important to note that the accuracy of cardiac echocardiography in assessing PAH pressures is limited and relies on the experience of the physician [3]. Due to the absence of straightforward diagnostic biomarkers for PAH, the diagnosis of PAH often lags behind the manifestation of clinical symptoms in patients. Findings from the REVEAL study revealed that 21.1% of 2967 patients exhibited symptoms of PAH two years before receiving a formal diagnosis [6]. The pursuit of diagnostic biomarkers for PAH is imperative to enhance early detection and prognosis for patients.

The inflammatory response plays a significant role in the progression of various disease processes. The pathogenesis of PH is a complex phenomenon involving the abnormal activity of endothelial cells, smooth muscle cells, and Fibroblasts. This leads to remodeling of the pulmonary vasculature. In the development of PAH, both structural and molecular changes take place in the pulmonary arteries, with a specific focus on extracellular matrix remodeling, inflammation, immune cell infiltration, metabolic imbalance, and the activation of signaling pathways [7]. Inflammation plays a crucial role in the development of PAH, particularly in pulmonary vascular remodeling. The intricate interplay between cytokines (such as IL-6, IL-1β, TNF, etc.) and immune cells (including Macrophages, T cells, and NK cells) further complicates the comprehension of the precise role of the inflammatory response in PAH [8,9]. Several ongoing clinical studies are currently focusing on therapeutic approaches that target inflammatory and immune-related pathways associated with PAH. These studies provide additional evidence of the potential of inflammation and immunity as novel avenues for the treatment of PAH [10,11]. By mining PAH-related hub genes from an inflammatory perspective, our study contributes to the discovery of both therapeutic targets and novel diagnostic markers for PAH. Furthermore, we construct diagnostic models for PAH and enhance clinical comprehension of this disease.

## 2. Materials and Methods

### 2.1. Data Retrieval and Processing

We searched the GEO database to identify relevant transcriptomic datasets and single-cell data pertaining to pulmonary hypertension patients. Specifically, we found four transcriptomic datasets (GSE113439, GSE117261, GSE15197, and GSE33463) as well as one single-cell dataset (GSE228644) [12,13,14,15,16]. All of the datasets included in this study consisted of both a pulmonary hypertension group and a control group. The sample numbers and sequencing platform information for each dataset are summarized in Table 1. However, for the experimental group in dataset GSE33463, which encompassed 20 samples from non-pulmonary arterial hypertension patients, these samples were excluded from the analysis in order to prevent any interference with the results. To begin, we merged the four transcriptomic datasets, extracted the common genes and their corresponding expression values for data filtration, and subsequently utilized this processed data as a new dataset for further analysis.

### 2.2. Dataset Merging

To address the inconsistencies among the platforms of the four datasets, we employed the removeBatchEffect function from the limma package in R software (version 4.2.3) to eliminate any batch effects. This step ensured the integration of the datasets into a unified and coherent analysis [17]. The batch correction and data normalization were performed using the normalizeBetweenArrays function.

### 2.3. Identification of PAH Subclasses

In order to identify subclasses of PAH, we obtained a list of inflammatory response-related genes (IRGs) from GeneCards (https://www.genecards.org/) and MSigDB databases (M5932, M8838, M13807, M15261, M41709, M41711, M41718, and M47215) [18]. These IRGs encompass a majority of the genes associated with the inflammatory response. Using the IRGs as an input gene set, we conducted consensus clustering through the ConsensusClusterPlus package to further categorize the PAH samples into subclasses [19]. For the cluster formation process, we defined the maximum number of clusters as 10 and the threshold of the cluster consensus score as 0.8.

### 2.4. Analysis of Immune Infiltration and Clinical Risk Gene Expression

To conduct the analysis, we employed a range of immune infiltration scores, such as ESTIMATE, CIBERSORT, XCELL, MCPcounter, quanTIseq, and EPIC, among others [20,21,22,23,24,25]. After conducting various analyses of immune infiltration, we observed the expression of different subclasses in immune cells following clustering. Regarding clinical risk correlation, previous studies have provided substantial evidence linking 12 genes (BMPR2, ACVRL1, ATP13A3, CAV1, EIF2AK4, ENG, GDF2, KCNK3, KDR, SMAD9, SOX17, and TBX4) to the development of PAH [26,27]. Of the 12 reported risk genes, we conducted further analysis of their expression within two identified subclasses, IRA and IRB, following clustering.

### 2.5. Weighted Gene Co-Expression Network Analysis (WGCNA) and Enrichment Analysis

WGCNA is a method used to study gene expression patterns across samples [28]. It helps group genes with similar expression, making it easier to see how these groups relate to certain traits. In our research, we used WGCNA to analyze gene expression data from two groups: IRA and IRB. First, we filtered the data to remove genes with low expression and removed any outlier samples. We then found the best soft threshold for our analysis, ensuring a strong scale-free network. With this, we built a co-expression network and grouped genes into modules based on their functions, using colors to distinguish them. Similar modules were combined for analysis. We identified significant genes by calculating Gene Significance (GS) and Module Membership (MM). Genes with |MM| > 0.5 and |GS| > 0.4 were selected for further study. To understand these genes better, we conducted enrichment analyses using specific databases. We focused on Gene Ontology (GO) to explore gene functions and the KEGG database to understand the roles of these genes in biological pathways [29].

### 2.6. Machine Learning Screening of Candidate Genes

After obtaining the candidate genes mentioned above, we employed machine learning techniques to screen for hub genes. To analyze the biological significance of these genes, we initially constructed a protein–protein interaction (PPI) network using the STRING (https://version-12-0.string-db.org, version: 12.0) database. Next, we utilized the CytoHubba plugin in the Cytoscape software (https://apps.cytoscape.org, version: 3.9.1) to rank the attributes of the node genes [30]. This refinement was achieved by employing a combination of 12 machine learning methods and 113 algorithmic approaches. These machine learning methods primarily include Lasso (least absolute shrinkage and selection operator), Stepglm, support vector machine (SVM), random forest (RF), Linear Discriminant Analysis (LDA), eXtreme Gradient Boosting (XGBoost), Gradient Boosting Machine (GBM), Ridge, elastic network (Enet), Generalized Linear Model Boosting (glmBoost), Stepglm, plsRglm, and NaiveBayes, among others. A comparison of the advantages and disadvantages of the 12 machine learning models can be found in Supplementary Table S1. For screening, we initially utilized GSE117261 as the training set for variable screening, employing cross-validation with one algorithm. Subsequently, we selected GSE113439, GSE15197, and GSE33463 as the training set for another algorithm to develop the classification prediction model. The size of the AUC (Area Under Curve) of the model served as the criterion for evaluating model accuracy. Moreover, to verify if the hub genes possess the ability to distinguish pulmonary hypertension disease from normal disease in a clinical setting, we employed box plots to further confirm the expression of the hub genes in the GSE117261 dataset and identified the genes with differential expression as the hub genes.

### 2.7. Expression of Hub Genes in the Single-Cell Dataset (GSE228644)

The single-cell dataset (GSE228644) was initially processed for quality control using the Seurat package (version: 4.3.0). Data points with low gene expression (nFeature_RNA ≥ 200 and ≤7500), high mitochondrial gene content (percent.mt ≤ 15), and high ribosomal gene content (percent.rb ≤ 15) were filtered out. After quality control, the data underwent sequential processing steps, including cluster removal, identification of highly variable genes, normalization, dimensionality reduction, and HARMONY de-clustering. T-SNE was used to visualize the clusters obtained after dimensionality reduction. The number of principal components (PCs) was determined using a combination of methods, including JackStraw, heatmap analysis, and ElbowPlot. FindNeighborhoods was used to select the appropriate resolution. The top five genes for each cluster were extracted using FindAllMarkers. Manual cell type annotation was performed for each cluster based on Crnkovic’s study, which integrated information from multiple databases (CellMark 2.0 and Cell Taxonomy), among others [12,31,32]. To investigate the involvement of IRGs in intercellular communication, we utilized the CellChat package to analyze the interaction of IRGs between cellular receptors and ligands [33].

### 2.8. Construction and Evaluation of the Diagnostic Model

To assess the diagnostic efficacy of the hub genes identified, diagnostic models were constructed using these genes. The regplot package was employed to generate diagnostic column line graphs and calibration curves for the hub genes. Logistic Regression was utilized to calculate the AUC curve, providing an evaluation of the model’s accuracy and the diagnostic value of the hub genes. In order to further evaluate the clinical utility of the model, clinical decision curves were drawn using the rmda package (version: 1.6), thus providing additional assessment of both the clinical value of the model and the hub genes.

### 2.9. Animal Experiments

In order to validate the actual expression level of the hub genes in PAH, we conducted animal experiments by modeling the disease using the widely reported dose of MCT drug (60 mg/kg). This dosage has been consistently used in the relevant literature [34]. Six-week-old Sprague Dawley (SD) rats (*n* = 3) were intraperitoneally injected with a dose of 60 mg/kg to establish the PAH group. The control group (*n* = 3) was given the same dose of saline. The SD rats were housed in a standard laboratory environment for 4 weeks with ad libitum access to water and food. The success of the model was confirmed by jugular vein cannulation to measure the systolic pressure of the right ventricle and by examination of lung tissue pathological sections for signs of pulmonary hypertension. This animal experiment was conducted with the approval of the Laboratory Animal Welfare Ethical Review of Beijing Anzhen Hospital Laboratory Animal Center, Capital Medical University (AZ2023LA010).

### 2.10. Quantitative Reverse-Transcription Polymerase Chain Reaction (qPCR)

For quantification of hub genes, rat lung tissues were stored at −80 °C. The Taq Pro Universal SYBR qPCR Master Mix kit (Q712-03) from Vazyme (Nanjing, China) was used to perform the qPCR assay. The 2x mix, forward primer, reverse primer, ddH2O, and cDNA were mixed and amplified following the instructions provided with the reagent. To ensure data reliability, all hub genes were analyzed three times. The primer sequences can be found in Supplementary Table S2.

### 2.11. Statistical Analysis

Statistical analysis was primarily conducted using R software (version 4.2.3) and GraphPad Prism (version 9.5). A significance level of *p* < 0.05 was adopted to determine statistical significance (ns indicates not significant, * *p* < 0.05, ** *p* < 0.01, *** *p* < 0.001, **** *p* < 0.0001).

## 3. Results

### 3.1. Classification of PAH Subtypes

**Figure 1:**
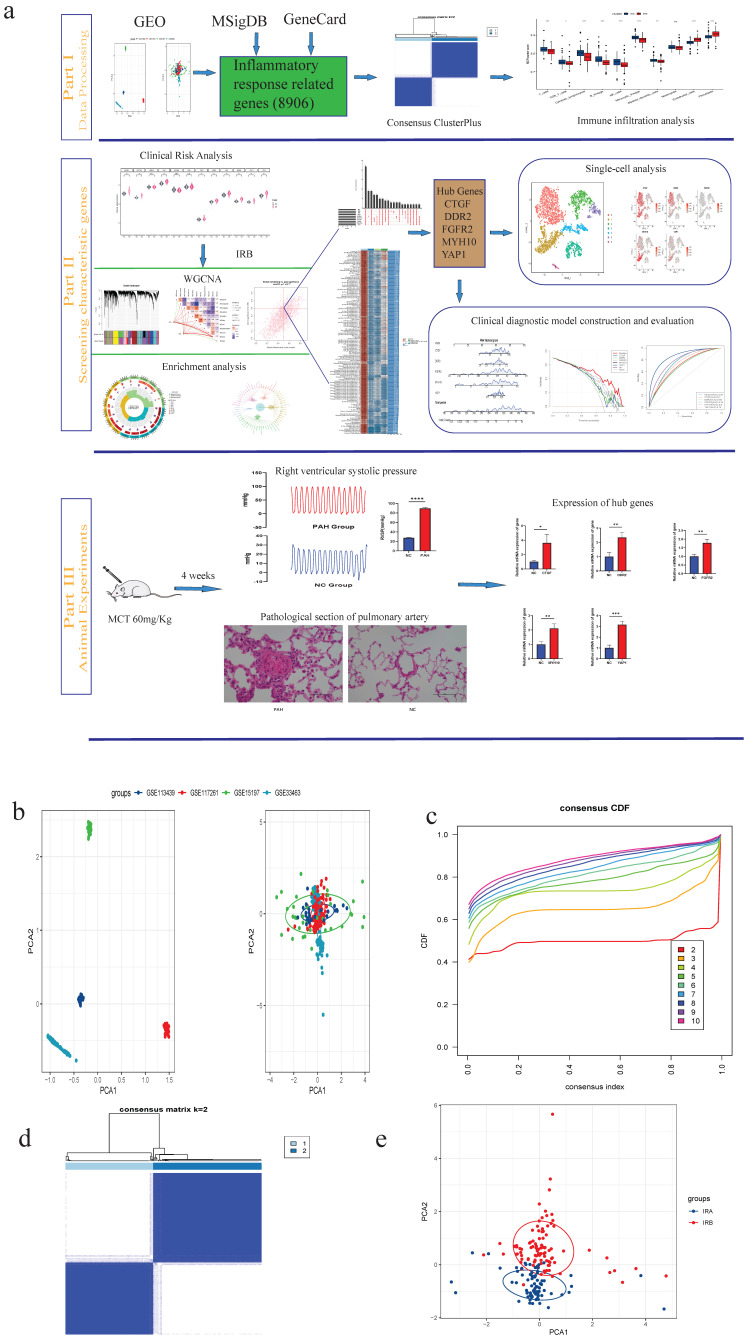
Classification of PAH subtypes. (**a**) Flowchart of the sturdy; Part I data processing; Part II screening for characteristic genes; Part III animal experiments. (**b**) Data set removal batch. (**c**) Consensus CDF of PAH. (**d**) Categorizing PAH into two subgroups. (**e**) Distribution of PCA in IRA and IRB (ns indicates not significant, * *p* < 0.05, ** *p* < 0.01, *** *p* < 0.001, **** *p* < 0.0001).

To better illustrate our work, we created a flowchart for this study (Figure 1a). We processed the transcriptome datasets (GSE113439, GSE117261, GSE15197, and GSE33463) to remove the batch effect. From the PCA (Principal Component Analysis) plot (Figure 1b), we observed that the batch effect between the four datasets was effectively eliminated, and they were merged into a single dataset suitable for subsequent data analysis. For our study, we obtained 2367 and 8529 IRGs from the GeneCard and MSigDB databases, respectively. After eliminating duplicate genes, we included a total of 8906 genes for further investigation. To visualize the cumulative distribution function (CDF) at different cluster numbers (κ), we employed a consensus CDF plot. Through a consistent clustering analysis, PAH subtypes were successfully classified into two to ten consecutive subtypes. The consensus CDF plot was utilized to visualize the different clusters (Supplementary Figure S1). Based on the observations from Figure 1c, it is evident that when κ = 2, the CDF curve is flatter, resulting in minimized fluctuation and maximized area under the curve. Therefore, we selected κ = 2 to categorize PAH into two subgroups, namely, IRA and IRB (Figure 1d). Furthermore, through PCA, we effectively demonstrated the distribution of IRGs between IRA and IRB in PAH, confirming the presence of two distinct subgroups (Figure 1e).

### 3.2. Immune Infiltration and Clinical Risk Gene Expression Analysis

**Figure 2:**
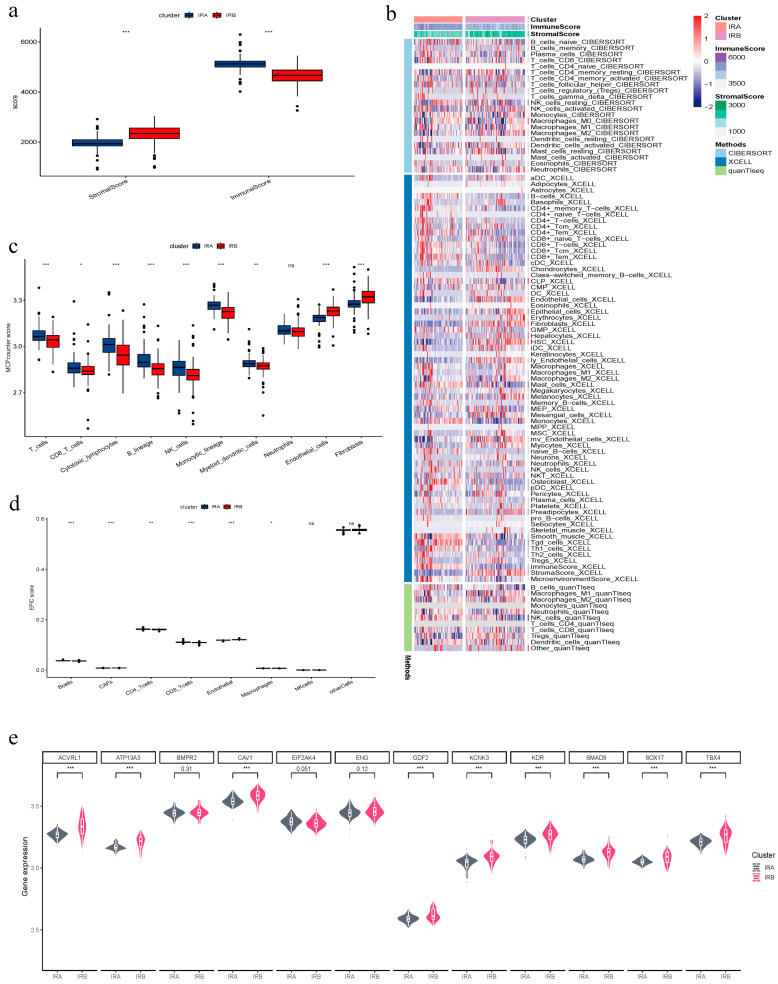
Immune Infiltration and Clinical Risk Gene Expression Analysis. (**a**) Immunescore and Stromalscore for subclasses of PAH. (**b**) Heatmap demonstration of CIBERSORT, XCELL, and quanTIseq methods. (**c**) Analysis of the MCPcounter algorithm for subclasses of PAH. (**d**) Analysis of the EPIC algorithm for subclasses of PAH. (**e**) Expression of risk genes in subclasses of PAH (ns indicates not significant, * *p* < 0.05, ** *p* < 0.01, *** *p* < 0.001).

To evaluate the association between two subclasses of PAH, specifically IRA and IRB, and immune cells, we conducted a series of analyses using the immune infiltration algorithm. Applying the ESTIMATE algorithm, we assessed the IRA and IRB subclasses in relation to both immune and stromal cells, which resulted in a calculated score. The analysis showed that IRA had a higher score for immunescore, whereas IRB exhibited a higher score for stromalscore (Figure 2a). In order to illustrate the disparities in the multi-group immune infiltration scores between the two groups (IRA and IRB), we combined the CIBERSORT, XCELL, and quanTIseq algorithms into a single heatmap for a more visually enhanced representation of the expression of IRA and IRB (Figure 2b). By employing the MCPcounter algorithm, an analysis revealed that in T cells, CD8 T cells, Cytotoxic lymphocytes, B lineage, NK cells, Monocytic lineage, and Myeloid dendritic cells, IRA exhibited higher MCPcounter scores compared to IRB. Conversely, in the scores for endothelial cells and Fibroblasts, IRA exhibited lower scores in contrast to IRB (Figure 2c). EPIC was also utilized for immune infiltration score analysis, which displayed differences between IRA and IRB in B cells, CAFs, CD4 T cells, CD8 T cells, endothelial cells, and Macrophages (Figure 2d). Both EPIC and MCPcounter algorithms demonstrated higher scores for IRA in CD8 T cells compared to IRB, whereas in terms of endothelial scores, IRB obtained higher scores compared to IRA. In terms of clinical risk, we utilized box plots to further illustrate the expression of 12 risk genes associated with the development of PAH disease (Figure 2e). From the box plots, we observed discrepancies in the expression of risk genes between IRA and IRB, with the exception of no differences in the expression of BMPR2, EIF2AK4, and ENG genes. Moreover, the expression levels of IRB were higher than those of IRA, thereby leading us to select IRB as a subcategory for the subsequent study of PAH.

### 3.3. WGCNA Analysis

**Figure 3:**
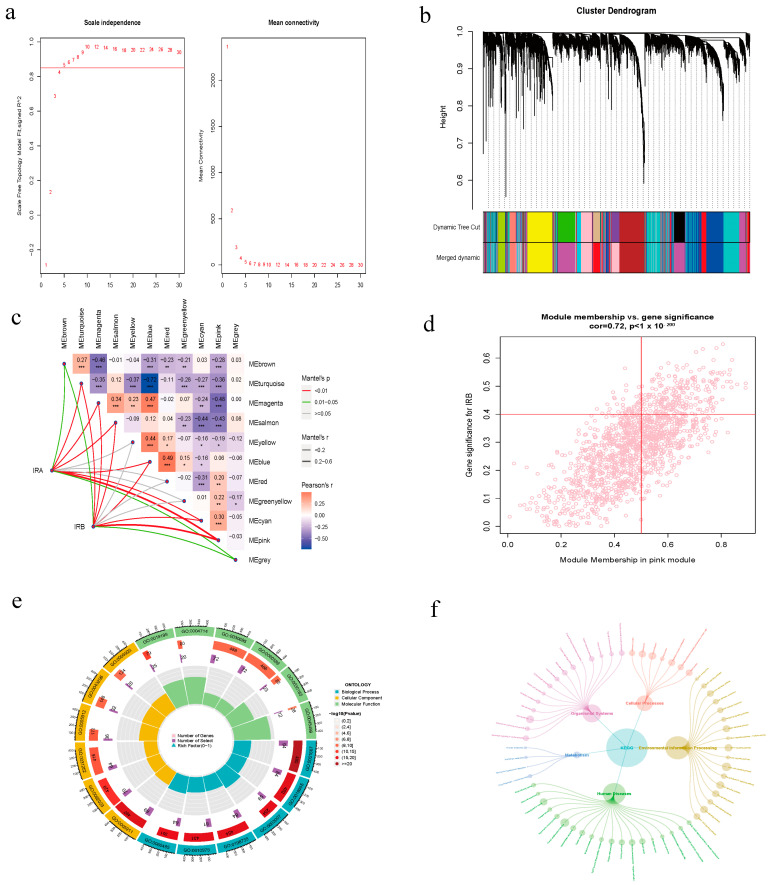
WGCNA Analysis. (**a**) Determine the soft threshold. (**b**) Merge similar gene modules. (**c**) Heatmap of correlation between modular genes and subclasses of PAH. (**d**) Pink module in correlation analysis with GS and MM. (**e**) GO enrichment analysis of pink module genes. (**f**) KEGG enrichment analysis of pink module genes (ns indicates not significant, * *p* < 0.05, ** *p* < 0.01, *** *p* < 0.001).

To identify genes related to IRB, we performed WGCNA analysis on the merged dataset. We first conducted cluster analysis, removing outlier samples (Supplementary Figure S2). After re-clustering, we set the optimal soft threshold (softPower = 5) from Figure 3a, establishing a scale-free co-expression network with 11 modules (Figure 3b), excluding gray modules as uninformative. The heatmap (Figure 3c) showed that the pink module had the strongest positive correlation with IRB. Correlation analysis indicated a strong relationship between IRB and the pink module (r = 0.72, *p* < 1× 10^−200^. We selected 216 hub genes based on |MM| > 0.5 and |GS| > 0.4, resulting in 1470 genes in the pink module (Figure 3d). GO and KEGG enrichment analyses revealed that pink module genes are mainly involved in the Wnt signaling pathway, neuron projection development, cell junction assembly, and PI3K–Akt signaling pathway, among others (Figure 3e,f). Detailed results can be found in Supplementary Tables S3 and S4.

### 3.4. Screening of Featured Genes Using Multiple Approaches and Machine Learning

**Figure 4:**
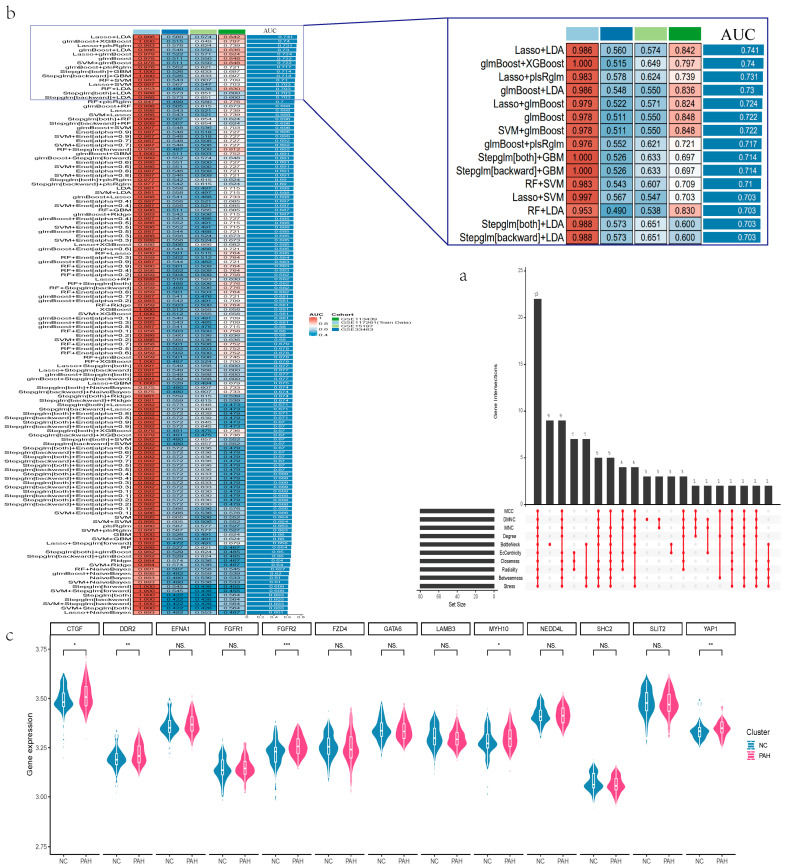
Screening of featured genes. (**a**) CytoHubba plugin screens upset plots of characterized genes. (**b**) AUC for multiple combinations of machine learning algorithms. (**c**) Differential expression of hub genes in PAH and NC (ns indicates not significant, * *p* < 0.05, ** *p* < 0.01, *** *p* < 0.001).

The 216 genes were utilized in an additional screening process to narrow down the candidate genes. This screening process involved the use of STRING and the CytoHubba plugin in Cytoscape software, which employed multiple algorithms. Among these algorithms, 22 genes were found to be significant (Figure 4a). To ensure the accuracy and robustness of the hub genes, we further assessed the candidate 22 genes using 12 machine learning methods in 113 algorithms. Among the combination of multiple machine learning algorithms, the Lasso + LDA approach yielded the highest AUC value (AUC = 0.741) (Figure 4b). Additionally, the complementary characteristics of Lasso and LDA, combined with the enhanced robustness and generalization ability of the model, contributed to its superior performance. This combination approach further reduced the number of mid-candidate genes from 22 to 13, which included CTGF, DDR2, EFNA1, FGFR1, FGFR2, FZD4, GATA6, LAMB3, MYH10, NEDD4L, SHC2, SLIT2, and YAP1. Next, we compared the expression levels of these candidate genes between the PAH and NC groups using box plots (Figure 4c). Our analysis revealed that only CTGF, DDR2, FGFR2, MYH10, and YAP1 displayed differential expression patterns between the two groups. Accordingly, we selected these genes as the hub genes to construct a diagnostic model.

### 3.5. Expression of Hub Genes in Single-Cell Dataset

**Figure 5:**
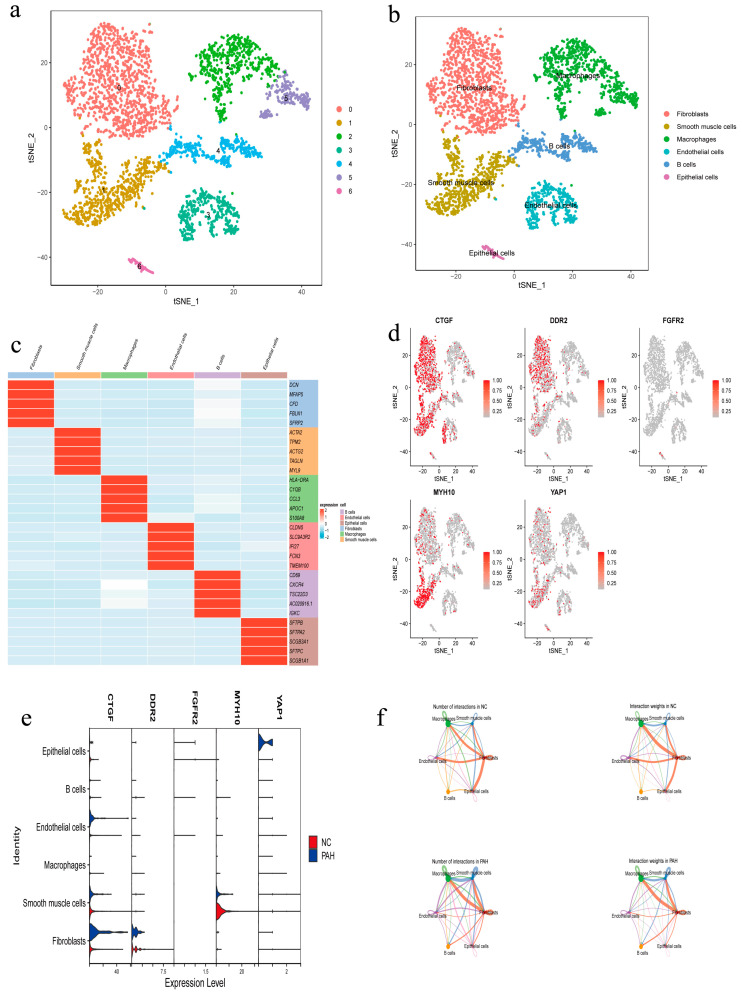
Expression of hub genes in single-cell dataset. (**a**) tSNE demonstrates the seven clusters identified. (**b**) Annotation of clusters. (**c**) Top five marker gene expression for each cell type. (**d**) Information on the distribution of hub genes in various cells. (**e**) Expression of hub genes between various cell types in PAH and NC. (**f**) Analysis of communication between different cells.

A total of 3672 cells were obtained after rigorous quality control of single-cell samples from three control (NC) and three experimental (PAH) groups (Supplementary Figures S3 and S4). Through subsequent processes such as batch removal and dimensionality reduction, we obtained a total of seven clusters (Figure 5a). Each cluster was manually annotated using various methods, including in the literature, CellMark2.0, and SingleR. The cell types identified were Fibroblasts, smooth muscle cells, Macrophages, endothelial cells, B cells, and Epithelial cells (Figure 5b). During the annotation process, we extracted the top five genes from each cluster and visualized them (Supplementary Table S5 and Supplementary Figure S5). A heatmap was used to demonstrate the expression of the top five marker genes in cell types (Figure 5c). To understand the distribution of the hub genes within the clusters, tSNE was used for downscaling to show their positional distribution (Figure 5d). Upon analysis, we found that CTGF and DDR2 were mainly distributed in Fibroblasts, MYH10 was primarily distributed in smooth muscle cells, YAP1 was present in both Fibroblasts and smooth muscle cells, and FGFR2 showed lower expression primarily concentrated in Epithelial cells.

To further illustrate the expression of the hub genes between the various cell types in the PAH and NC groups, we visualized their expression using violin plots (Figure 5e). CTGF expression was higher in Fibroblasts of the PAH group compared to the NC group. Furthermore, we performed cell–cell communication analysis to understand the information exchange between these six cell types, considering both the number of inter-communication interactions and the interaction weights (Figure 5f). We discovered that the communication strength between Fibroblasts and Macrophages, as well as smooth muscle cells in the PAH group, was enhanced in terms of both the number of interactions and the weight of interactions compared to the NC group. Conversely, the communication strength between Fibroblasts and Epithelial cells in the PAH group was weakened in both the number and weight of interactions.

### 3.6. Construction of Clinical Diagnostic Model

**Figure 6:**
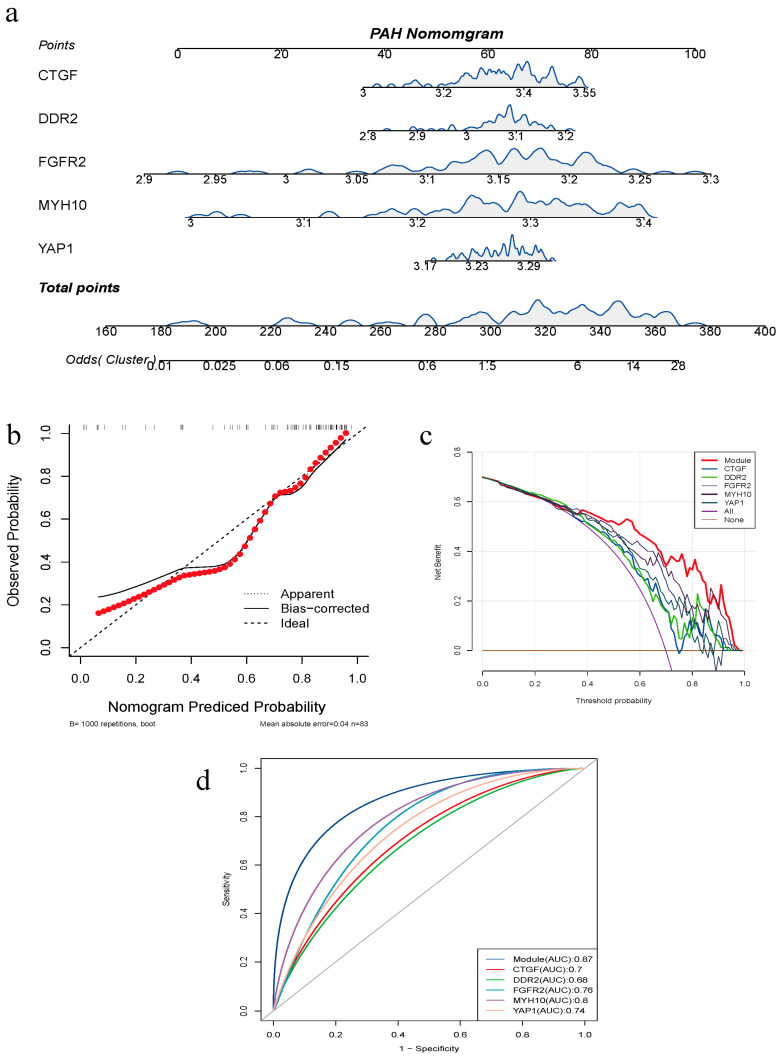
Construction of clinical diagnostic model. (**a**) Construction of PAH nomograms. (**b**) Calibration curves for assessing the predictive power of nomograms. (**c**) Decision curves for assessing the clinical value of nomograms. (**d**) ROC evaluation of hub genes and models.

To construct the nomograms for PAH (Figure 6a), we selected five genes for screening. By calibrating the curve, we observed a consistent accuracy probability of PAH compared to the predicted probability (Figure 6b). The clinical decision curve provides valuable insights into the model’s performance in a clinical setting. Analyzing the DCA curves, we found that the model consistently outperforms both “intervention for none” and “intervention for all” when the threshold value exceeds approximately 0.2 (Figure 6c). Moreover, when the threshold value surpasses 0.4, both the individual genes and the overall model demonstrate higher risk prediction than “intervention for none” and “intervention for all”. Overall, the model displays excellent assessment capabilities. To assess its accuracy, we further evaluated the model’s performance and the diagnostic efficacy of the hub genes through ROC curves (Figure 6d). The constructed diagnostic model exhibited high accuracy (AUC = 0.87). Among the individual genes, MYH10 displayed the highest diagnostic efficacy (AUC = 0.8), while DDR2 exhibited the lowest diagnostic efficacy (AUC = 0.68).

### 3.7. Expression of Hub Genes in Animal Tissues

**Figure 7:**
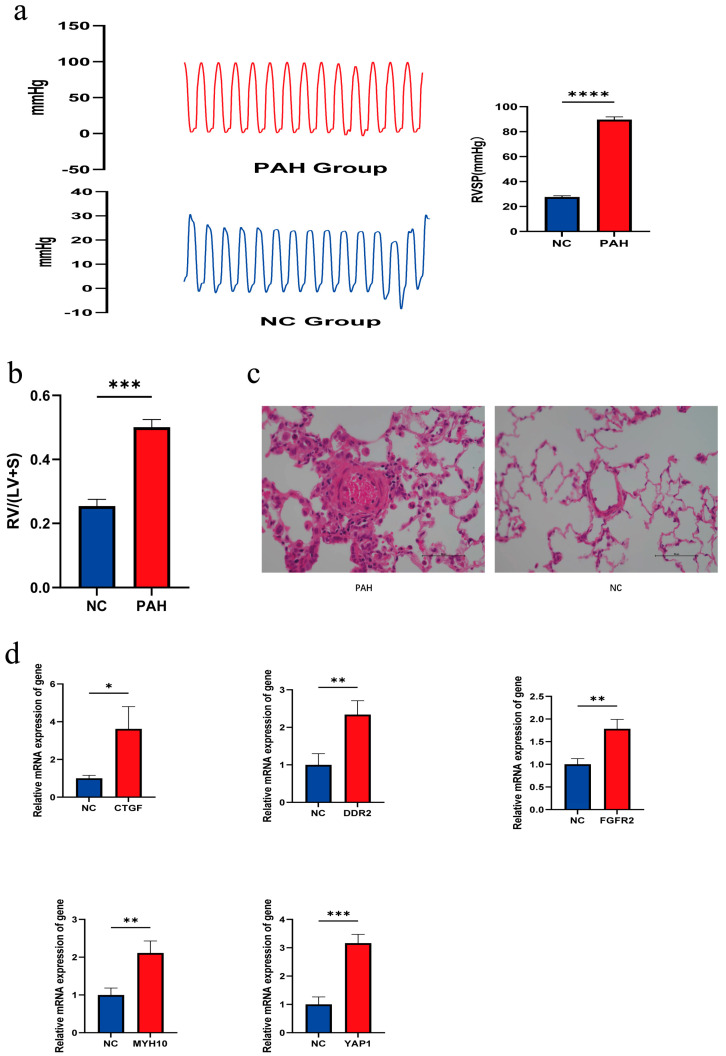
Expression of hub genes in animal tissues. (**a**) Comparison of RVSP. (**b**) Cardiac hypertrophy index between PAH and NC groups. (**c**) Vascular remodeling in PAH and NC lung tissue. (**d**) Expression of hub genes (ns indicates not significant, * *p* < 0.05, ** *p* < 0.01, *** *p* < 0.001, **** *p* < 0.0001).

The expression of hub genes in animal tissues was investigated in this study. Primer sequences for the hub gene were designed and the expression was verified in SD rats using lung tissues obtained from the animal model of PAH. To validate the successful construction of the PAH animal model, the right ventricular systolic pressure (RVSP) and cardiac hypertrophy index (RV/LV + S) were examined in the PAH group (Supplementary Figure S6). Comparative analysis revealed significantly higher RVSP (Figure 7a) and cardiac hypertrophy index (Figure 7b) in the PAH group compared to the control group. Lung pathology sections (Figure 7c) further confirmed the successful construction of the PAH animal model. Additionally, mRNA expression levels of CTGF, DDR2, FGFR2, MYH10, and YAP1 genes were analyzed by quantitative polymerase chain reaction (qPCR) and found to be higher in the PAH group than in the control group (Figure 7d), which was consistent with the previous analysis.

## 4. Discussion

Pulmonary hypertension is a rare yet extremely perilous disease characterized by an increase in pulmonary artery pressure. Its pathomechanism involves severe remodeling of the pulmonary vasculature. This, in turn, exacerbates the burden on the right ventricle, potentially leading to heart failure. When pulmonary vascular remodeling manifests in PAH, it is accompanied by perivascular inflammation and infiltration of inflammatory cells. Additionally, there is a correlation between perivascular inflammation and the extent of pulmonary vascular remodeling [35]. Research has demonstrated that IRGs are involved in the pathogenesis of systemic sclerosis-associated PAH by modulating T cell activity, thereby promoting disease progression [36]. The onset of PAH is often insidious and characterized by a lack of specific diagnostic biomarkers. The current literature indicates that proteomics, metabolomics, and genomics significantly contribute to the identification of biomarkers for PAH [37,38,39]. Machine learning holds significant promise for biomarker screening and is predominantly applied in the analysis of clinical data associated with PAH [40,41,42,43,44]. The inflammatory response is a fundamental and critical pathophysiological mechanism underlying PAH. Consequently, we employed a range of machine learning algorithms from an inflammatory standpoint to investigate potential biomarkers of IRGs in the genomics of PAH.

We conducted an analysis on the transcriptome dataset by merging it with another dataset, creating a new dataset. Initially, we constructed an expression matrix that focused on IRGs. Consensus CDF analysis was performed on the PAH group, leading to the classification of two subclasses: IRA and IRB. WGCNA was then used to identify candidate genes associated with IRB. To determine the hub genes, we employed 12 machine learning algorithms to create 113 different models. Through this process, we identified 8 hub genes (CTGF, DDR2, EFNA1, FGFR1, FGFR2, GATA6, MYH10, and YAP1). To gain further insight into the role of these hub genes, we examined their expression profiles in PAH cell subtypes using single-cell datasets.

Connective tissue growth factor (CTGF), also known as CCN2, is a secreted protein synthesized by umbilical vein and vascular endothelial cells. It plays a crucial role in the production of the extracellular matrix, as well as cell adhesion and other essential functions in different cell types, including smooth muscle cells, Fibroblasts, and osteoblasts [45]. CTGF plays a pivotal role in the promotion of fibroblast differentiation into myofibroblasts, facilitating the deposition of extracellular matrix collagen and triggering tissue fibrosis [46]. The absence of CTGF greatly diminishes pulmonary vascular remodeling and right ventricular hypertrophy in a well-established animal model of PH [47]. Discoidin domain receptor 2 (DDR2) is a member of the Discoidin domain receptor family, which consists of receptor tyrosine kinases involved in cell adhesion. DDR2 primarily participates in collagen activation and plays significant roles in fibrosis and cellular proliferation. Type I collagen has the ability to activate the MMP-2 gene expression through DDR2, thereby stimulating fibroblast migration [48]. In the context of lung fibrosis, DDR2 synergistically interacts with TGF-β and fibrillar collagen to facilitate the conversion of Fibroblasts into myofibroblasts, as well as the upregulation of vascular endothelial growth factor. Experimental studies employing animal models have demonstrated that the downregulation of DDR2 can mitigate the development of lung fibrosis [49]. Fibroblast growth factor receptor 2 (FGFR2) displays elevated expression levels in hypoxic pulmonary hypertension. In this context, endothelial-derived FGFR2 plays a crucial role in sustaining an aberrant endothelial cell phenotype through autocrine secretion [50]. Under hypoxic conditions, FGFR in endothelial cells serve as regulators of the Endothelial-to-Mesenchymal Transition (EndMT) response, thereby inducing PH [51]. The coding product of the MYH10 gene is an actin whose main function is to participate in the construction of the cytoskeleton. Kim et al. [52] discovered that MYH10 plays a crucial role in the maintenance of extracellular matrix stability in the lungs, primarily by interacting with THBS and MMP10. The absence of MYH10 results in disruptions in the myosin network, preventing the secretion of THBS. Consequently, this alteration affects the activity of MMP10, ultimately leading to extracellular matrix remodeling. Yes-associated protein (YAP) is a pivotal transcription factor in the Hippo pathway. The Hippo–YAP1/TAZ axis plays a crucial role in modulating cell proliferation, differentiation, and apoptosis, thereby influencing target organs [53]. One of the primary pathological features of PAH is the excessive accumulation of endothelial cells and smooth muscle cells in the lung. Studies have revealed that Galectin-3 (Gal-3) can regulate the YAP/FOXM1/cyclinD1 signaling pathway, thereby controlling the proliferation of smooth muscle cells in the pulmonary artery [54]. In this study, we focused on screening the hub genes that directly or indirectly impact the development of PH. These genes are implicated in extracellular matrix-associated fibrosis (CTGF, DDR2, MYH10, and YAP1), cell proliferation (DDR2), and endothelial cell function (FGFR2). Altered remodeling of the extracellular matrix is an important feature of PH disease, with collagen deposition having the most pronounced effect on the endothelium, an important feature of PH vascular remodeling [55]. A substantial body of evidence supports the notion that immune cells influence cellular mechanisms through various means, including cell-to-cell interactions, secretion of inflammatory cytokines and chemokines, and modulation of extracellular matrix-associated enzymes. As a result, these immune-mediated processes significantly contribute to the vascular remodeling observed in PH.

There is now ample evidence that immune cells can impact vascular remodeling in PH through various mechanisms, such as cell-to-cell interactions, secretion of inflammatory cytokines and chemokines, and modulation of extracellular matrix-associated enzymes [56]. The results of our single-cell analysis demonstrate an increase in the number of interactions and the strength of communication of interaction weights between Fibroblasts, Macrophages, and smooth muscle cells in the PAH group. The secretion of IL-6 by activated Fibroblasts may potentially influence Macrophage and extracellular matrix remodeling in PAH [56]. The IL-6 pathway plays a significant role in PAH. However, a recent study by Toshner et al. [57] demonstrated that the IL-6 receptor antagonist Tocilizumab did not lead to improvements in pulmonary vascular resistance, possibly indicating its limited effectiveness for a specific type of PAH. Nonetheless, the complex and intricate involvement of immune cells in PAH should not overshadow the fact that they remain a promising therapeutic target for the treatment of PAH [56].

Limitations: In this study, we conducted analyses using transcriptomic and single-cell data. However, the sample size was limited, and differences in populations, ethnicities, regions, and the heterogeneity of PAH have hindered broader sample validation. Consequently, the accuracy of the identified biomarkers and models requires further validation across diverse populations, ethnicities, and PAH subtypes. Considering the imperfections of different machine learning algorithms, the high AUC value obtained in this study does not rule out the risk of overfitting. Additionally, the validation conducted in this study primarily relied on animal models, with limited support from clinical samples. Future research will need to incorporate large clinical cohorts to comprehensively assess the diagnostic efficacy of these biomarkers. These limitations highlight the need for broader validation efforts to enhance the generalizability of the models and their clinical applicability.

While this study identifies potential biomarkers using machine learning models, ethical concerns like false positives (leading to unnecessary treatments) and false negatives (delaying treatment) remain critical. Adhering to the principle of Primum non nocere is essential to prevent harm from diagnostic uncertainty [58]. Model designs must address these risks, and future research should prioritize large-scale clinical validation. This will help ensure patient safety, minimize overdiagnosis or underdiagnosis, and create more reliable clinical tools.

## 5. Conclusions

This study investigates the pivotal genes associated with PAH from the standpoint of inflammation. The findings will facilitate the identification of potential therapeutic targets and novel diagnostic biomarkers for PAH through the examination of aspects of inflammation and immunity. By employing diverse machine learning techniques, our objective is to develop a diagnostic model for PAH and advance comprehension of this disease in clinical settings. While the screening of biomarkers can provide more early diagnostic information, we must recognize its limitations and take multiple measures to reduce the risks of false positives or false negatives.

## Figures and Tables

**Table 1:** Data information.

GEO Datasets	Platforms	PAH	NC
GSE113439	GPL6244	15	11
GSE117261	GPL6480	58	25
GSE15197	GPL6480	26	13
GSE33463	GPL6947	79	41
GSE228644	GPL20301	3	3

## Data Availability

All data can be obtained from the GEO database (https://www.ncbi.nlm.nih.gov/geo/, accessed on 15 December 2023).

## Supplementary Materials

The following supporting information can be downloaded at: https://www.mdpi.com/article/10.3390/diagnostics14212398/s1, Figure S1: consensus CDF plot for cluster visualization, Figure S2: removal of outliers, Figure S3: quality control of single-cell data, Figure S4: feature distribution plot of single-cell RNA sequencing data, Figure S5: violin plot of Top five gene expression, Figure S6: assessment of RVH in PAH and NC groups; Table S1: a comparison of the advantages and disadvantages of the 12 machine learning models, Table S2: primer sequence information, Table S3: information on GO enrichment analysis, Table S4: information on KEGG enrichment analysis, Table S5: Top five differential gene expression across clusters.

## References

[1] Humbert M., Kovacs G., Hoeper M.M., Badagliacca R., Berger R.M.F., Brida M., Carlsen J., Coats A.J.S., Escribano-Subias P., Ferrari P. (2022). 2022 ESC/ERS Guidelines for the diagnosis and treatment of pulmonary hypertension: Developed by the task force for the diagnosis and treatment of pulmonary hypertension of the European Society of Cardiology (ESC) and the European Respiratory Society (ERS). Endorsed by the International Society for Heart and Lung Transplantation (ISHLT) and the European Reference Network on rare respiratory diseases (ERN-LUNG). Eur. Heart J..

[2] Mocumbi A., Humbert M., Saxena A., Jing Z.C., Sliwa K., Thienemann F., Archer S.L., Stewart S. (2024). Pulmonary hypertension. Nat. Rev. Dis. Primers.

[3] Ruopp N.F., Cockrill B.A. (2022). Diagnosis and Treatment of Pulmonary Arterial Hypertension: A Review. JAMA.

[4] Ullah W., Minalyan A., Saleem S., Nadeem N., Abdullah H.M., Abdalla A., Chan V., Saeed R., Khan M., Collins S. (2020). Comparative accuracy of non-invasive imaging versus right heart catheterization for the diagnosis of pulmonary hypertension: A systematic review and meta-analysis. Int. J. Cardiol. Heart Vasc..

[5] Ni J.R., Yan P.J., Liu S.D., Hu Y., Yang K.H., Song B., Lei J.Q. (2019). Diagnostic accuracy of transthoracic echocardiography for pulmonary hypertension: A systematic review and meta-analysis. BMJ Open.

[6] Brown L.M., Chen H., Halpern S., Taichman D., McGoon M.D., Farber H.W., Frost A.E., Liou T.G., Turner M., Feldkircher K. (2011). Delay in recognition of pulmonary arterial hypertension: Factors identified from the REVEAL Registry. Chest.

[7] Zahid K.R., Raza U., Chen J., Raj U.J., Gou D. (2020). Pathobiology of pulmonary artery hypertension: Role of long non-coding RNAs. Cardiovasc. Res..

[8] Thenappan T., Ormiston M.L., Ryan J.J., Archer S.L. (2018). Pulmonary arterial hypertension: Pathogenesis and clinical management. BMJ.

[9] Soon E., Holmes A.M., Treacy C.M., Doughty N.J., Southgate L., Machado R.D., Trembath R.C., Jennings S., Barker L., Nicklin P. (2010). Elevated levels of inflammatory cytokines predict survival in idiopathic and familial pulmonary arterial hypertension. Circulation.

[10] Zamanian R.T., Badesch D., Chung L., Domsic R.T., Medsger T., Pinckney A., Keyes-Elstein L., D’Aveta C., Spychala M., White R.J. (2021). Safety and Efficacy of B-Cell Depletion with Rituximab for the Treatment of Systemic Sclerosis-associated Pulmonary Arterial Hypertension: A Multicenter, Double-Blind, Randomized, Placebo-controlled Trial. Am. J. Respir. Crit. Care Med..

[11] Trankle C.R., Canada J.M., Kadariya D., Markley R., De Chazal H.M., Pinson J., Fox A., Van Tassell B.W., Abbate A., Grinnan D. (2019). IL-1 Blockade Reduces Inflammation in Pulmonary Arterial Hypertension and Right Ventricular Failure: A Single-Arm, Open-Label, Phase IB/II Pilot Study. Am. J. Respir. Crit. Care Med..

[12] Crnkovic S., Valzano F., Fließer E., Gindlhuber J., Thekkekara P.H., Basil M., Morley M.P., Katzen J., Gschwandtner E., Klepetko W. (2022). Single-cell transcriptomics reveals skewed cellular communication and phenotypic shift in pulmonary artery remodeling. JCI Insight.

[13] Stearman R.S., Bui Q.M., Speyer G., Handen A., Cornelius A.R., Graham B.B., Kim S., Mickler E.A., Tuder R.M., Chan S.Y. (2019). Systems Analysis of the Human Pulmonary Arterial Hypertension Lung Transcriptome. Am. J. Respir. Cell Mol. Biol..

[14] Mura M., Cecchini M.J., Joseph M., Granton J.T. (2019). Osteopontin lung gene expression is a marker of disease severity in pulmonary arterial hypertension. Respirology.

[15] Cheadle C., Berger A.E., Mathai S.C., Grigoryev D.N., Watkins T.N., Sugawara Y., Barkataki S., Fan J., Boorgula M., Hummers L. (2012). Erythroid-specific transcriptional changes in PBMCs from pulmonary hypertension patients. PLoS ONE.

[16] Rajkumar R., Konishi K., Richards T.J., Ishizawar D.C., Wiechert A.C., Kaminski N., Ahmad F. (2010). Genomewide RNA expression profiling in lung identifies distinct signatures in idiopathic pulmonary arterial hypertension and secondary pulmonary hypertension. Am. J. Physiol.-Heart Circ. Physiol..

[17] Ritchie M.E., Phipson B., Wu D., Hu Y., Law C.W., Shi W., Smyth G.K. (2015). limma powers differential expression analyses for RNA-sequencing and microarray studies. Nucleic Acids Res..

[18] Liberzon A., Subramanian A., Pinchback R., Thorvaldsdóttir H., Tamayo P., Mesirov J.P. (2011). Molecular signatures database (MSigDB) 3.0. Bioinformatics.

[19] Wilkerson M.D., Hayes D.N. (2010). ConsensusClusterPlus: A class discovery tool with confidence assessments and item tracking. Bioinformatics.

[20] Racle J., Gfeller D. (2020). EPIC: A Tool to Estimate the Proportions of Different Cell Types from Bulk Gene Expression Data. Methods Mol. Biol..

[21] Finotello F., Mayer C., Plattner C., Laschober G., Rieder D., Hackl H., Krogsdam A., Loncova Z., Posch W., Wilflingseder D. (2019). Molecular and pharmacological modulators of the tumor immune contexture revealed by deconvolution of RNA-seq data. Genome Med..

[22] Li B., Liu J.S., Liu X.S. (2017). Revisit linear regression-based deconvolution methods for tumor gene expression data. Genome Biol..

[23] Becht E., Giraldo N.A., Lacroix L., Buttard B., Elarouci N., Petitprez F., Selves J., Laurent-Puig P., Sautès-Fridman C., Fridman W.H. (2016). Estimating the population abundance of tissue-infiltrating immune and stromal cell populations using gene expression. Genome Biol..

[24] Newman A.M., Liu C.L., Green M.R., Gentles A.J., Feng W., Xu Y., Hoang C.D., Diehn M., Alizadeh A.A. (2015). Robust enumeration of cell subsets from tissue expression profiles. Nat. Methods.

[25] Yoshihara K., Shahmoradgoli M., Martínez E., Vegesna R., Kim H., Torres-Garcia W., Treviño V., Shen H., Laird P.W., Levine D.A. (2013). Inferring tumour purity and stromal and immune cell admixture from expression data. Nat. Commun..

[26] Welch C.L., Aldred M.A., Balachandar S., Dooijes D., Eichstaedt C.A., Gräf S., Houweling A.C., Machado R.D., Pandya D., Prapa M. (2023). Defining the clinical validity of genes reported to cause pulmonary arterial hypertension. Genet. Med. Off. J. Am. Coll. Med. Genet..

[27] Rhodes C.J., Batai K., Bleda M., Haimel M., Southgate L., Germain M., Pauciulo M.W., Hadinnapola C., Aman J., Girerd B. (2019). Genetic determinants of risk in pulmonary arterial hypertension: International genome-wide association studies and meta-analysis. Lancet Respir. Med..

[28] Langfelder P., Horvath S. (2008). WGCNA: An R package for weighted correlation network analysis. BMC Bioinform..

[29] Wu T., Hu E., Xu S., Chen M., Guo P., Dai Z., Feng T., Zhou L., Tang W., Zhan L. (2021). clusterProfiler 4.0: A universal enrichment tool for interpreting omics data. Innovation.

[30] Chin C.H., Chen S.H., Wu H.H., Ho C.W., Ko M.T., Lin C.Y. (2014). cytoHubba: Identifying hub objects and sub-networks from complex interactome. BMC Syst. Biol..

[31] Jiang S., Qian Q., Zhu T., Zong W., Shang Y., Jin T., Zhang Y., Chen M., Wu Z., Chu Y. (2023). Cell Taxonomy: A curated repository of cell types with multifaceted characterization. Nucleic Acids Res..

[32] Hu C., Li T., Xu Y., Zhang X., Li F., Bai J., Chen J., Jiang W., Yang K., Ou Q. (2023). CellMarker 2.0: An updated database of manually curated cell markers in human/mouse and web tools based on scRNA-seq data. Nucleic Acids Res..

[33] Jin S., Guerrero-Juarez C.F., Zhang L., Chang I., Ramos R., Kuan C.H., Myung P., Plikus M.V., Nie Q. (2021). Inference and analysis of cell-cell communication using CellChat. Nat. Commun..

[34] Boucherat O., Agrawal V., Lawrie A., Bonnet S. (2022). The Latest in Animal Models of Pulmonary Hypertension and Right Ventricular Failure. Circ. Res..

[35] Rajagopal S., Yu Y.R. (2023). Determining the Architecture of Inflammation in Pulmonary Arterial Hypertension. Am. J. Respir. Crit. Care Med..

[36] Tu J., Jin J., Chen X., Sun L., Cai Z. (2022). Altered Cellular Immunity and Differentially Expressed Immune-Related Genes in Patients with Systemic Sclerosis-Associated Pulmonary Arterial Hypertension. Front. Immunol..

[37] Yokokawa T., Boucherat O., Martineau S., Lemay S.-E., Breuils-Bonnet S., Krishna V., Kalyana-Sundaram S., Jeyaseelan J., Potus F., Bonnet S. (2024). Prognostic Significance of Proteomics-Discovered Circulating Inflammatory Biomarkers in Patients with Pulmonary Arterial Hypertension. J. Am. Heart Assoc..

[38] Mismetti V., Delavenne X., Montani D., Bezzeghoud S., Delezay O., Hodin S., Launay D., Marchand-Adam S., Nunes H., Ollier E. (2023). Proteomic biomarkers for survival in systemic sclerosis-associated pulmonary hypertension. Respir. Res..

[39] Sen P., Shashikadze B., Flenkenthaler F., Van de Kamp E., Tian S., Meng C., Gigl M., Fröhlich T., Merkus D. (2023). Proteomics- and Metabolomics-Based Analysis of Metabolic Changes in a Swine Model of Pulmonary Hypertension. Int. J. Mol. Sci..

[40] Wang D., Huang S., Cao J., Feng Z., Jiang Q., Zhang W., Chen J., Kutty S., Liu C., Liao W. (2024). A comprehensive study on machine learning models combining with oversampling for bronchopulmonary dysplasia-associated pulmonary hypertension in very preterm infants. Respir. Res..

[41] Sweatt A.J., Hedlin H.K., Balasubramanian V., Hsi A., Blum L.K., Robinson W.H., Haddad F., Hickey P.M., Condliffe R., Lawrie A. (2019). Discovery of Distinct Immune Phenotypes Using Machine Learning in Pulmonary Arterial Hypertension. Circ. Res..

[42] Rhodes C.J., Sweatt A.J., Maron B.A. (2022). Harnessing Big Data to Advance Treatment and Understanding of Pulmonary Hypertension. Circ. Res..

[43] Dawes T.J.W., de Marvao A., Shi W., Fletcher T., Watson G.M.J., Wharton J., Rhodes C.J., Howard L.S.G.E., Gibbs J.S.R., Rueckert D. (2017). Machine Learning of Three-dimensional Right Ventricular Motion Enables Outcome Prediction in Pulmonary Hypertension: A Cardiac MR Imaging Study. Radiology.

[44] Nemati N., Burton T., Fathieh F., Gillins H.R., Shadforth I., Ramchandani S., Bridges C.R. (2024). Pulmonary Hypertension Detection Non-Invasively at Point-of-Care Using a Machine-Learned Algorithm. Diagnostics.

[45] Bradham D.M., Igarashi A., Potter R.L., Grotendorst G.R. (1991). Connective tissue growth factor: A cysteine-rich mitogen secreted by human vascular endothelial cells is related to the SRC-induced immediate early gene product CEF-10. J. Cell Biol..

[46] Wang X., Cui H., Wu S. (2019). CTGF: A potential therapeutic target for Bronchopulmonary dysplasia. Eur. J. Pharmacol..

[47] Tam A.Y.Y., Horwell A.L., Trinder S.L., Khan K., Xu S., Ong V., Denton C.P., Norman J.T., Holmes A.M., Bou-Gharios G. (2021). Selective deletion of connective tissue growth factor attenuates experimentally-induced pulmonary fibrosis and pulmonary arterial hypertension. Int. J. Biochem. Cell Biol..

[48] Ruiz P.A., Jarai G. (2012). Discoidin domain receptors regulate the migration of primary human lung fibroblasts through collagen matrices. Fibrogenesis Tissue Repair.

[49] Zhao H., Bian H., Bu X., Zhang S., Zhang P., Yu J., Lai X., Li D., Zhu C., Yao L. (2016). Targeting of Discoidin Domain Receptor 2 (DDR2) Prevents Myofibroblast Activation and Neovessel Formation During Pulmonary Fibrosis. Mol. Ther. J. Am. Soc. Gene Ther..

[50] Tu L., Dewachter L., Gore B., Fadel E., Dartevelle P., Simonneau G., Humbert M., Eddahibi S., Guignabert C. (2011). Autocrine fibroblast growth factor-2 signaling contributes to altered endothelial phenotype in pulmonary hypertension. Am. J. Respir. Cell Mol. Biol..

[51] Woo K.V., Shen I.Y., Weinheimer C.J., Kovacs A., Nigro J., Lin C.Y., Chakinala M., Byers D.E., Ornitz D.M. (2021). Endothelial FGF signaling is protective in hypoxia-induced pulmonary hypertension. J. Clin. Investig..

[52] Kim H.T., Yin W., Jin Y.J., Panza P., Gunawan F., Grohmann B., Buettner C., Sokol A.M., Preussner J., Guenther S. (2018). Myh10 deficiency leads to defective extracellular matrix remodeling and pulmonary disease. Nat. Commun..

[53] Lee M., Goraya N., Kim S., Cho S.H. (2018). Hippo-yap signaling in ocular development and disease. Dev. Dyn..

[54] Zhang Q., Li W., Zhu Y., Wang Q., Zhai C., Shi W., Feng W., Wang J., Yan X., Chai L. (2021). Activation of AMPK inhibits Galectin-3-induced pulmonary artery smooth muscle cells proliferation by upregulating hippo signaling effector YAP. Mol. Cell. Biochem..

[55] Jandl K., Marsh L.M., Hoffmann J., Mutgan A.C., Baum O., Bloch W., Thekkekara-Puthenparampil H., Kolb D., Sinn K., Klepetko W. (2020). Basement Membrane Remodeling Controls Endothelial Function in Idiopathic Pulmonary Arterial Hypertension. Am. J. Respir. Cell Mol. Biol..

[56] Jandl K., Radic N., Zeder K., Kovacs G., Kwapiszewska G. (2023). Pulmonary vascular fibrosis in pulmonary hypertension—The role of the extracellular matrix as a therapeutic target. Pharmacol. Ther..

[57] Toshner M., Church C., Harbaum L., Rhodes C., Villar Moreschi S.S., Liley J., Jones R., Arora A., Batai K., Desai A.A. (2022). Mendelian randomisation and experimental medicine approaches to interleukin-6 as a drug target in pulmonary arterial hypertension. Eur. Respir. J..

[58] Gravesteijn B.Y., Steyerberg E.W., Lingsma H.F. (2022). Modern Learning from Big Data in Critical Care: Primum Non Nocere. Neurocrit Care.

