# Supplementary material for: Exploring IRGs as a Biomarker of Pulmonary Hypertension Using Multiple Machine Learning Algorithms
Source: Diagnostics (Basel). 2024 Oct 28;14(21):2398. doi: 10.3390/diagnostics14212398 (PMC11545203; doi:10.3390/diagnostics14212398)
Supplement: Supplementary file 1 [file diagnostics-14-02398-s001.zip › Figure S1.pdf]

consensus matrix legend

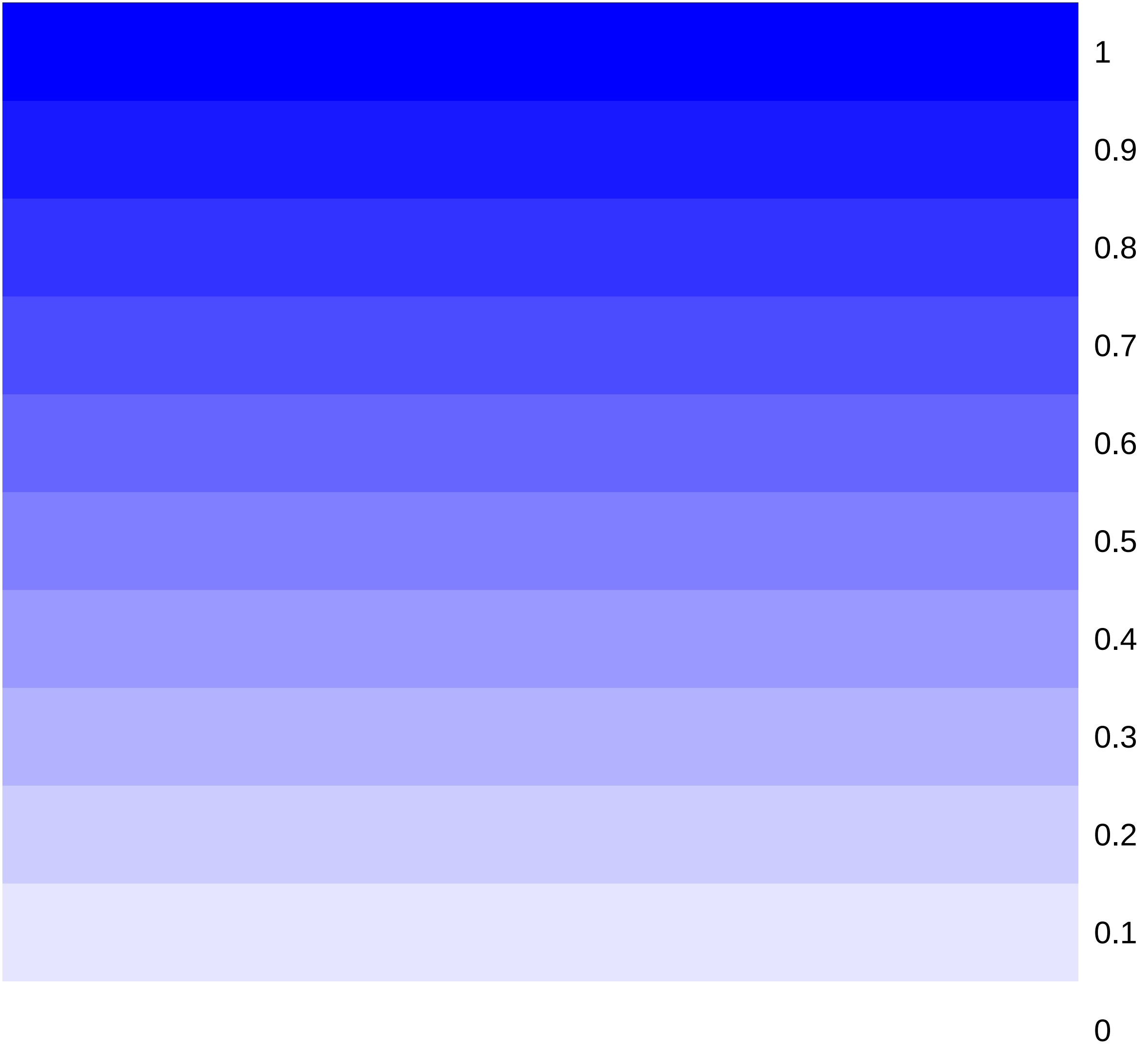

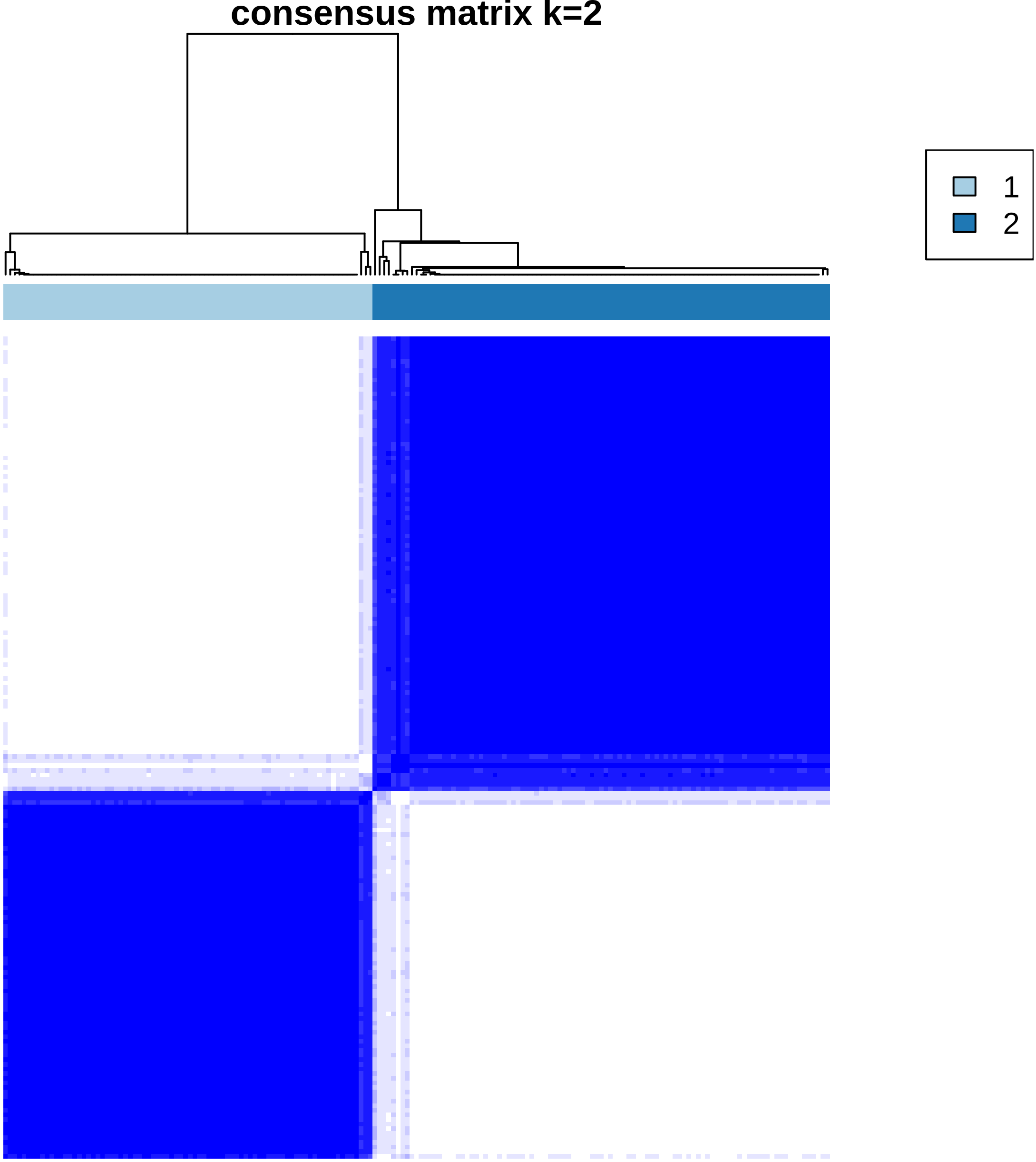
consensus matrix k=2
1
2

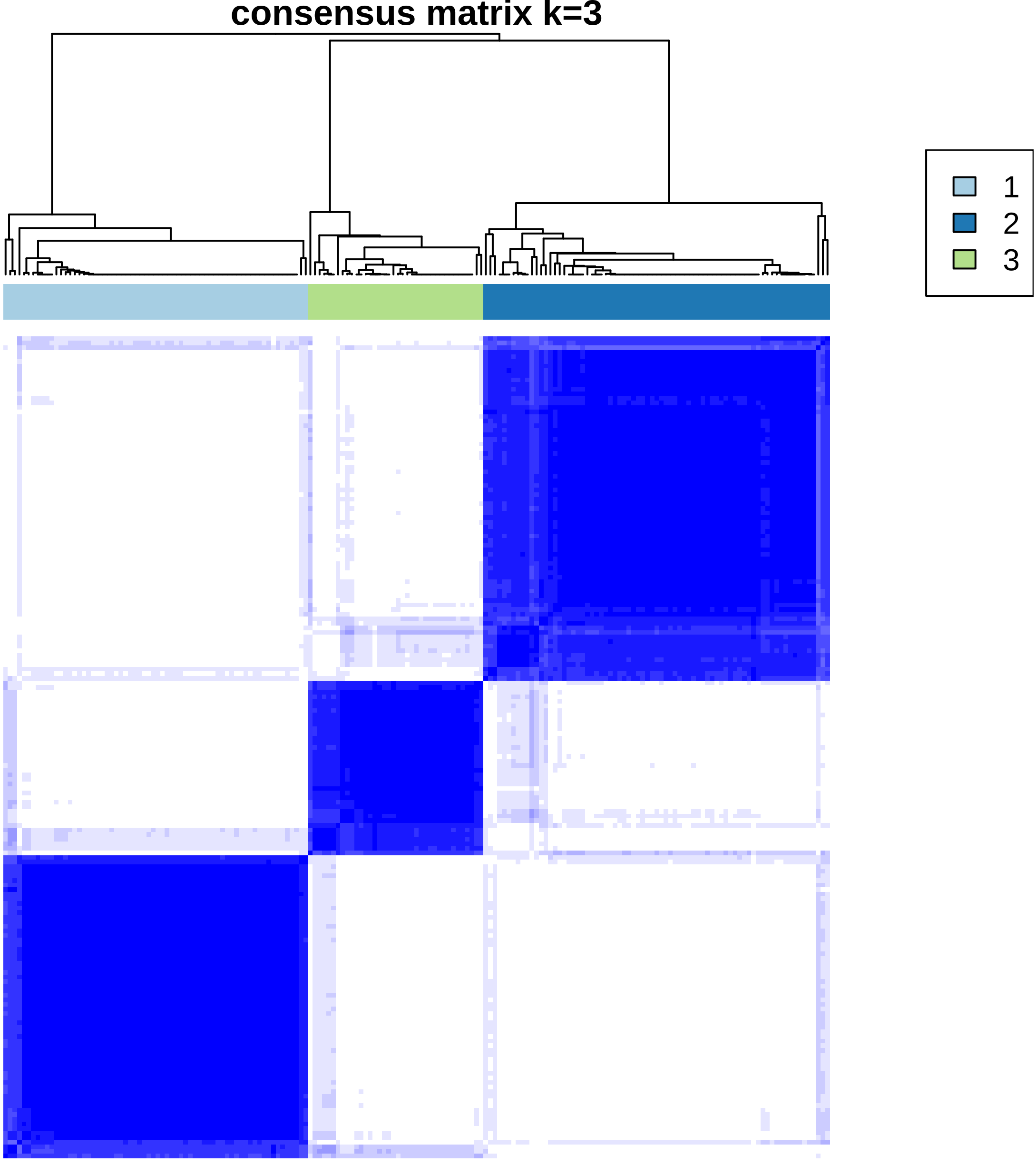
consensus matrix k=3
1
2
3

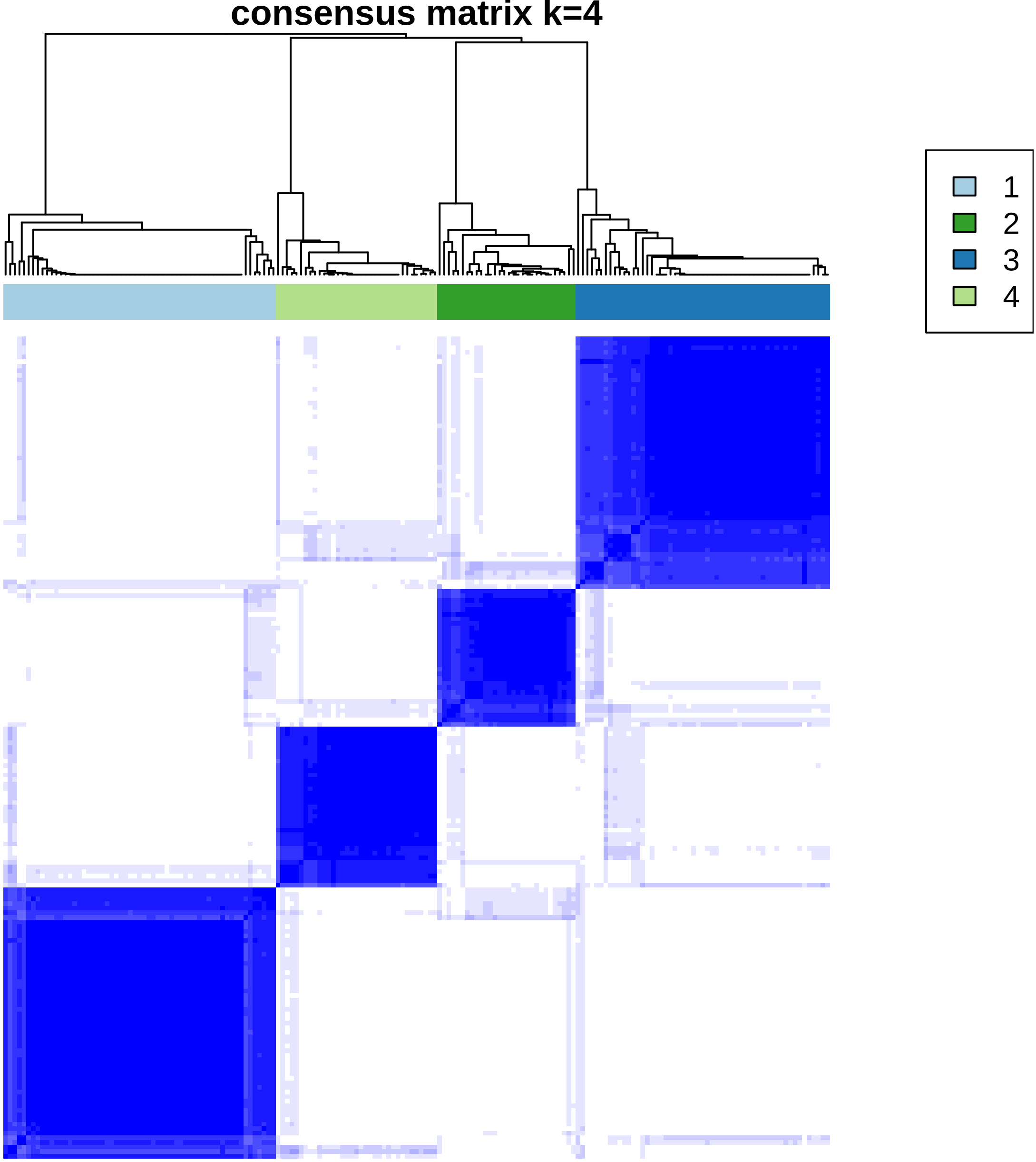
consensus matrix k=4
1
2
3
4

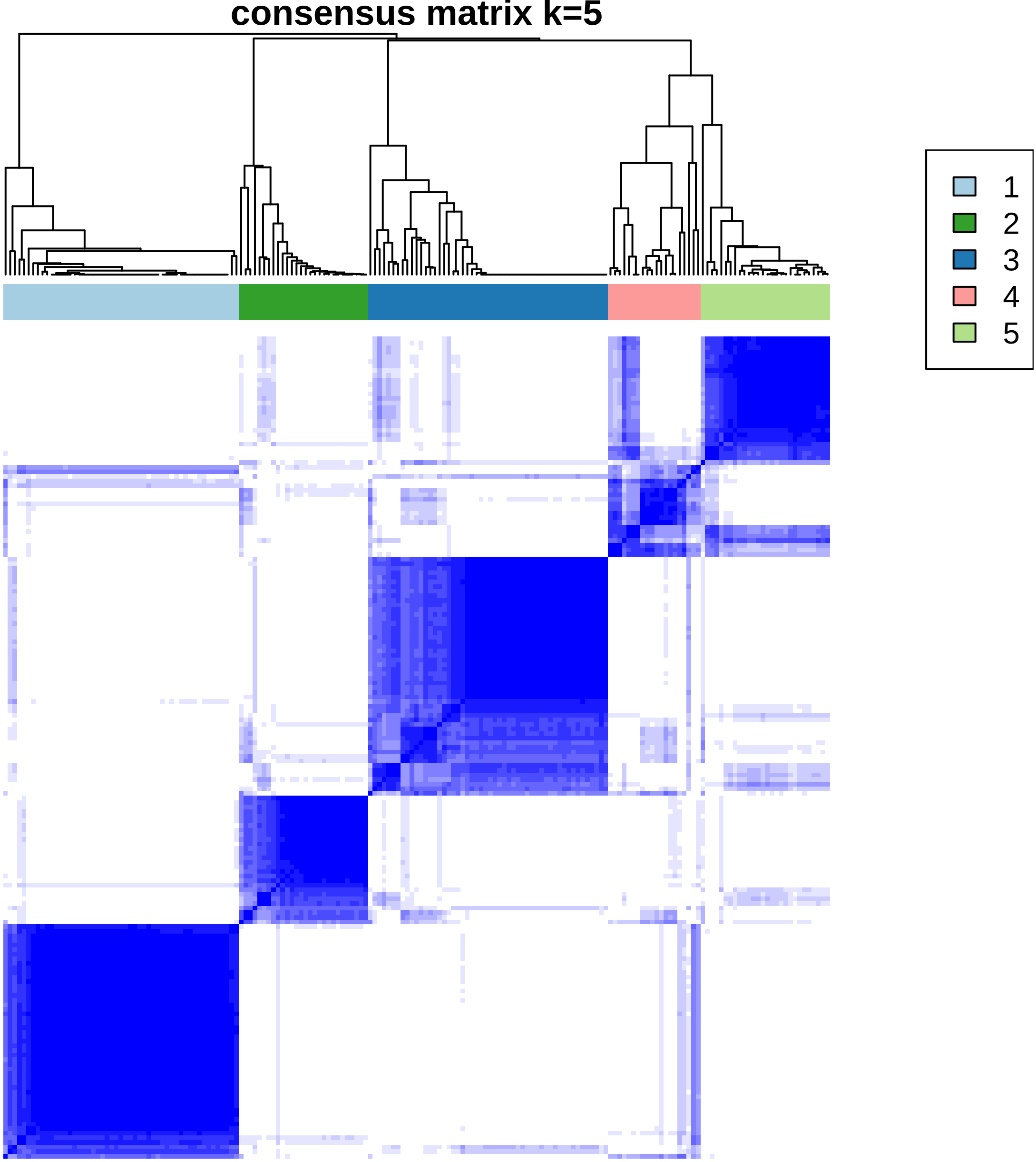
consensus matrix k=5
1
2
3
4
5

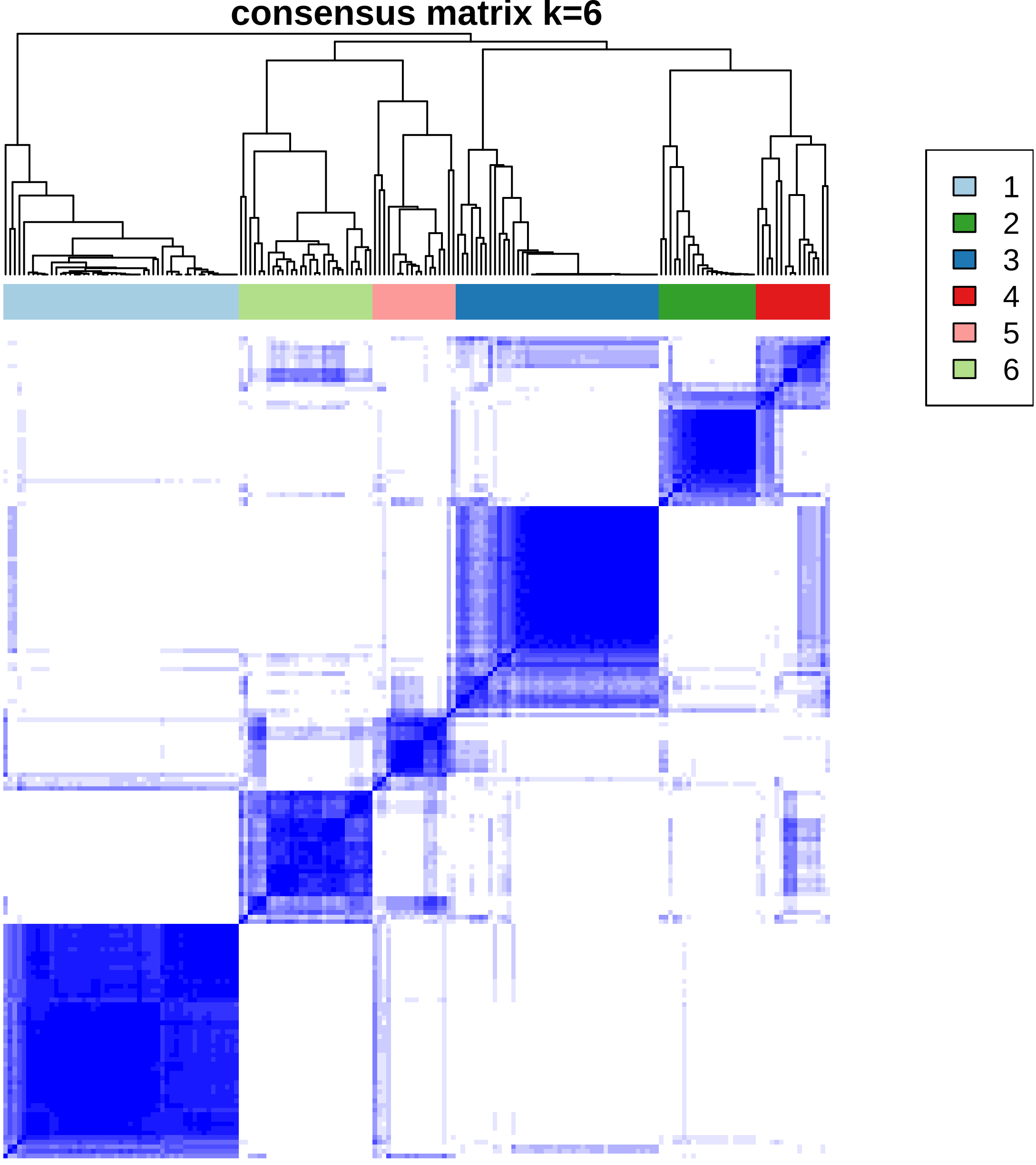
consensus matrix k=6
1
2
3
4
5
6

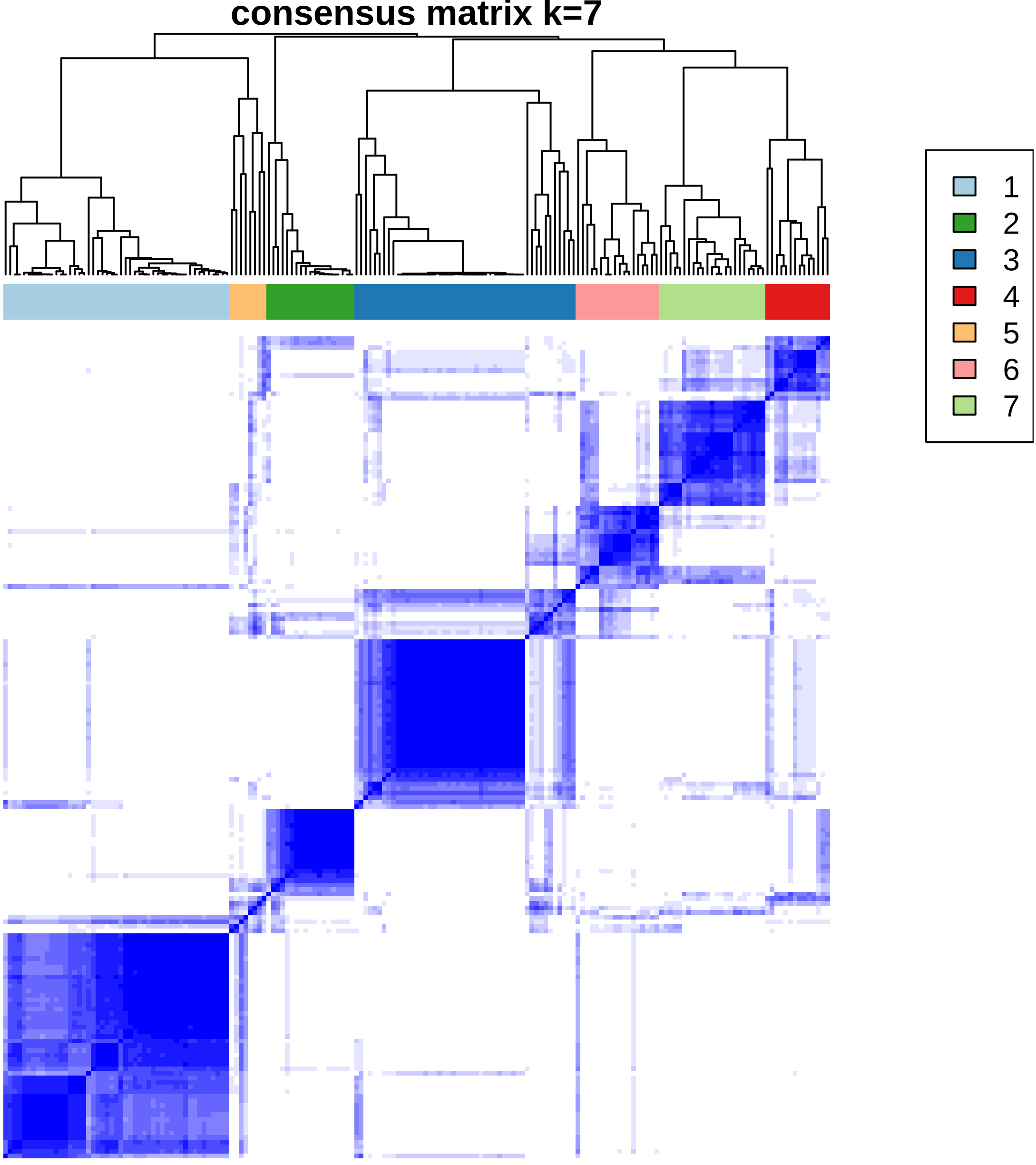
consensus matrix k=7
1
2
3
4
5
6
7

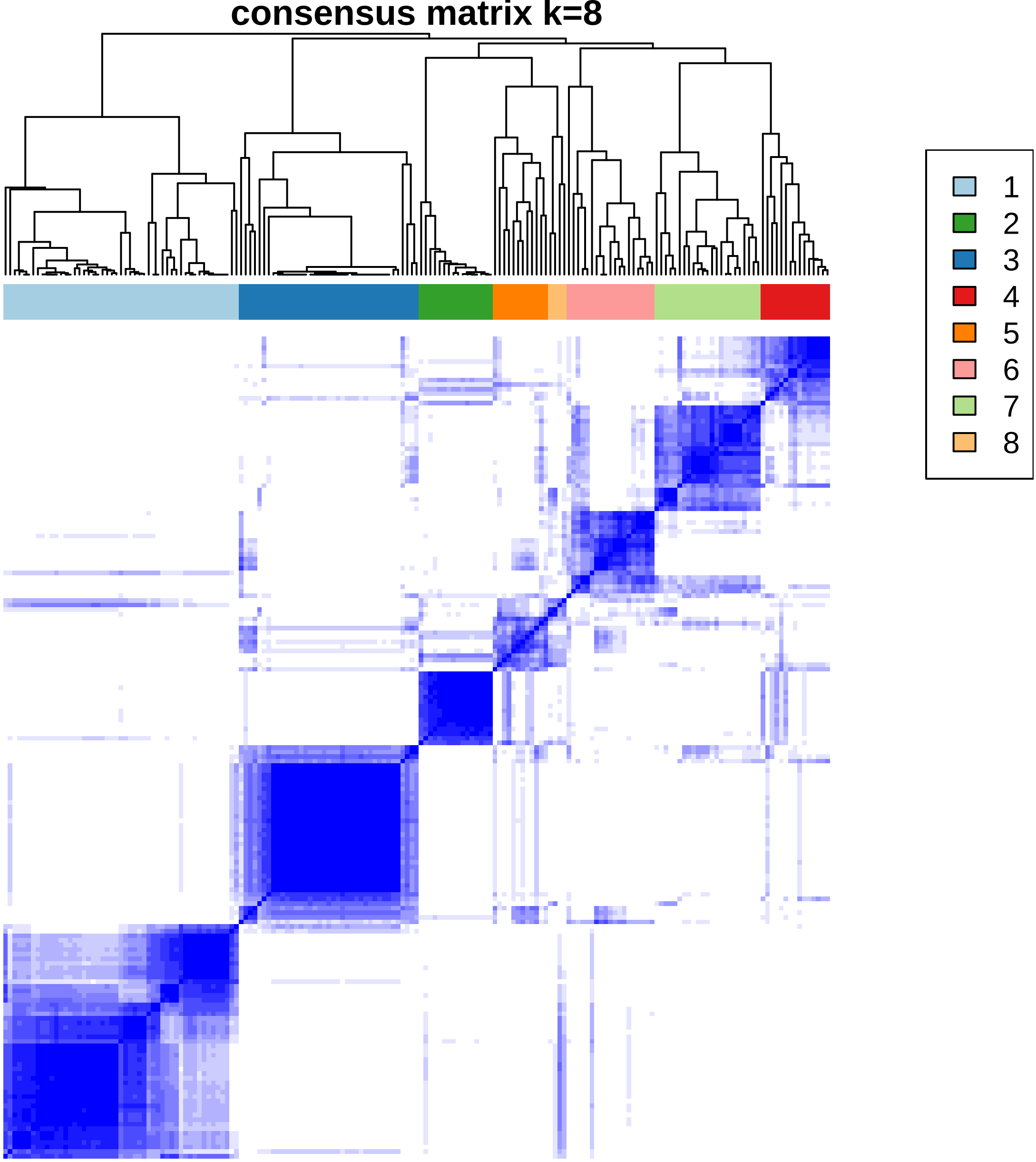
consensus matrix k=8
1
2
3
4
5
6
7
8

consensus matrix k=9

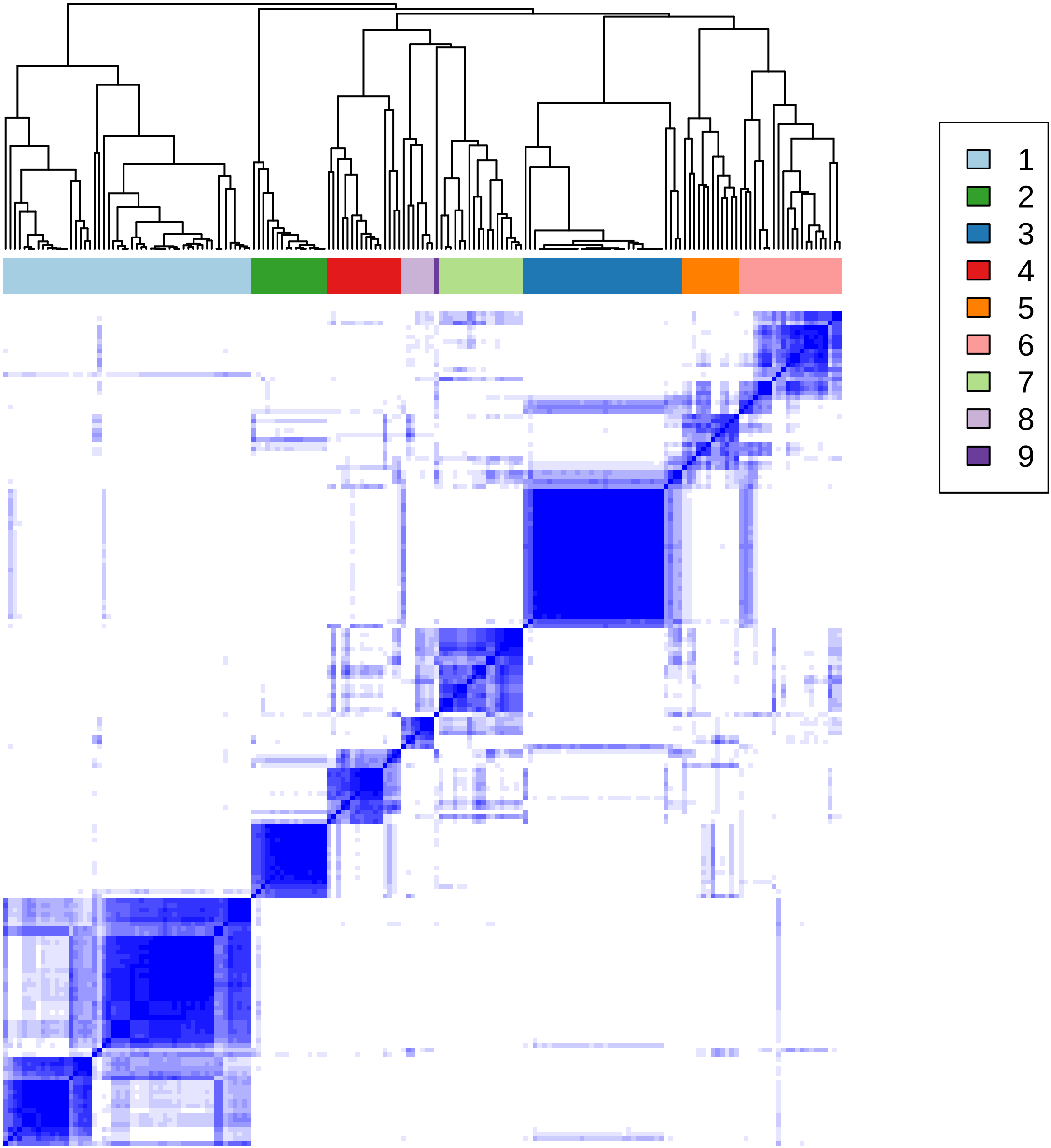

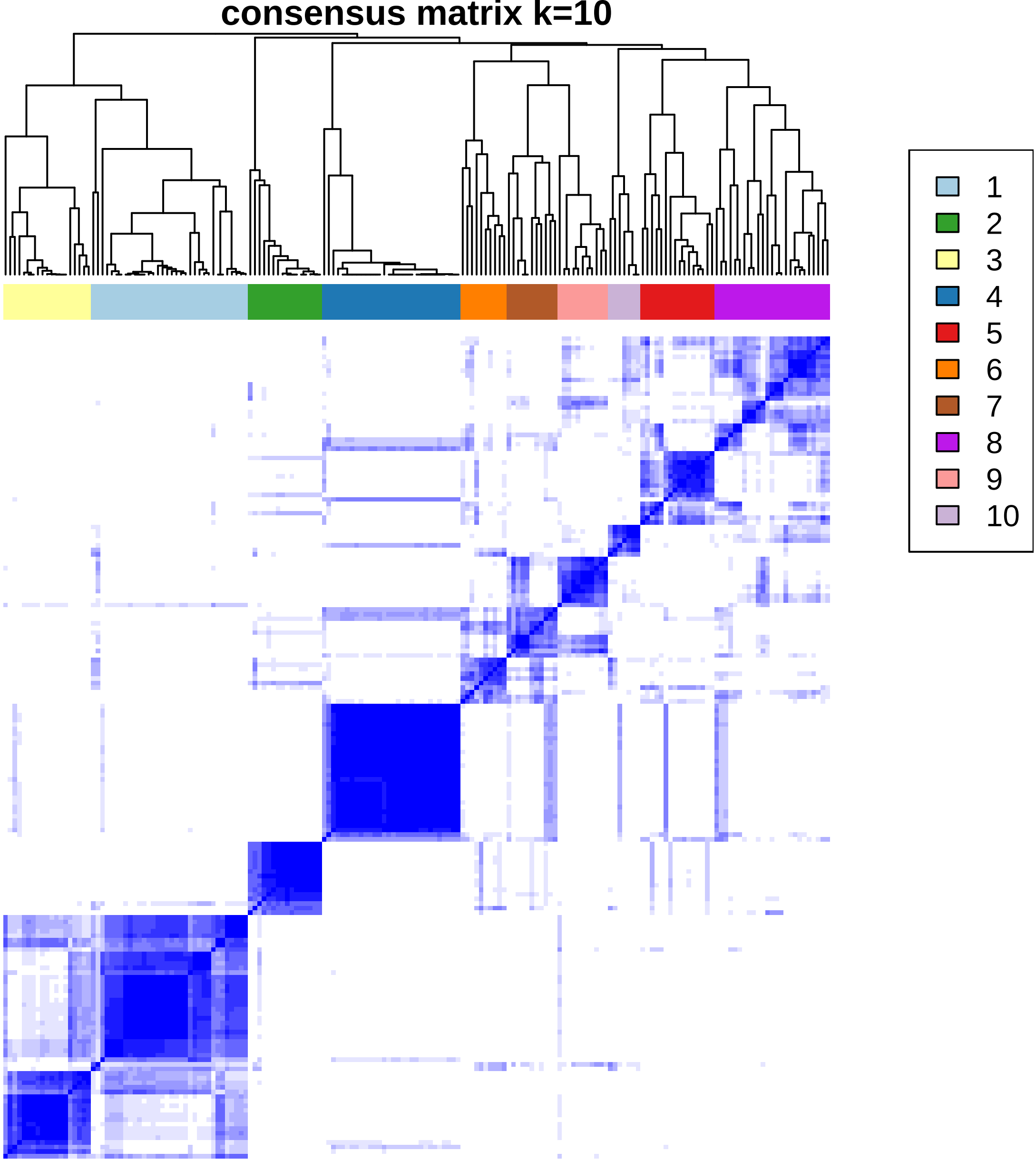
consensus matrix k=10
1
2
3
4
5
6
7
8
9
10

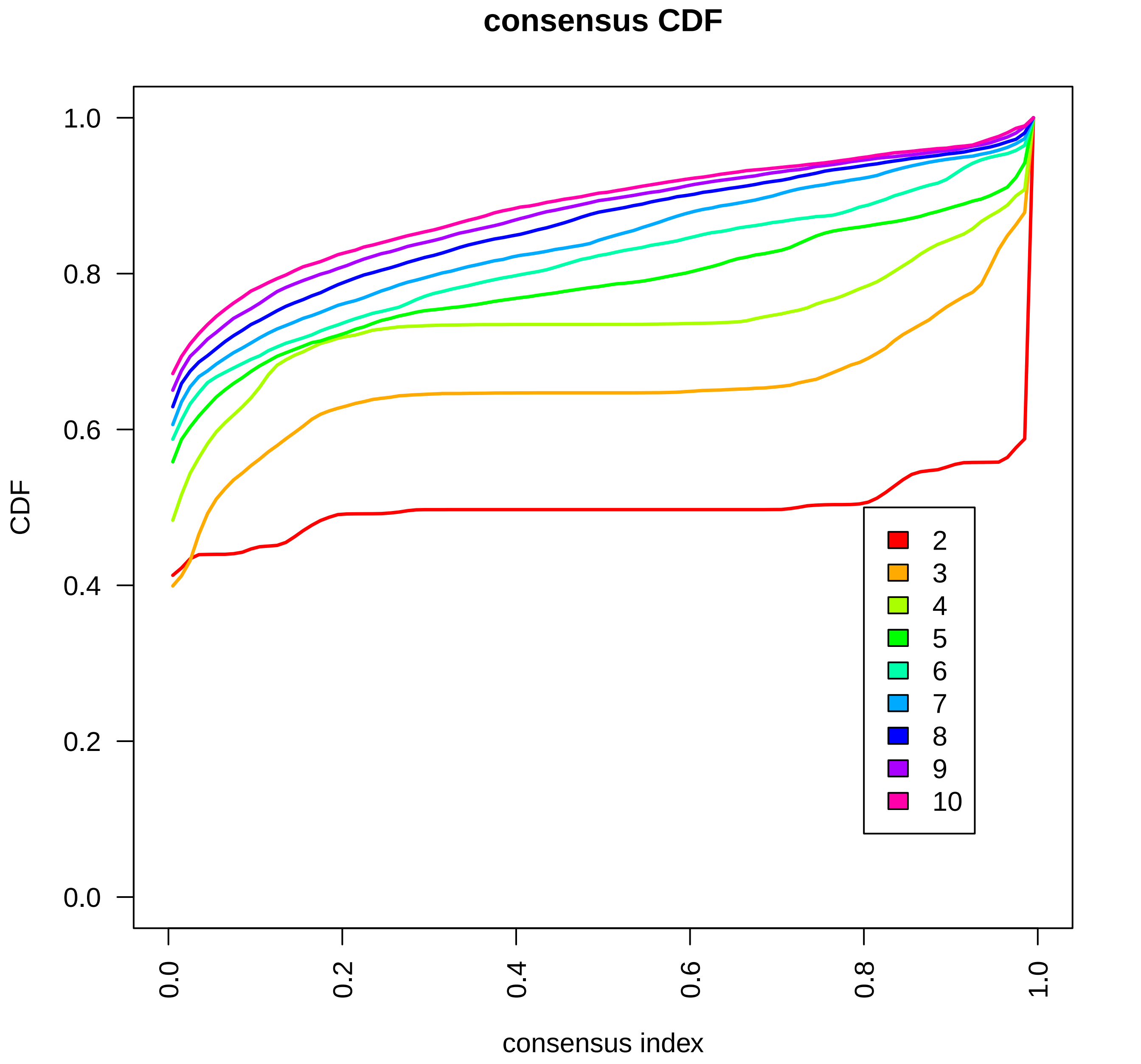
consensus CDF
CDF
consensus index
1.0
0.8
0.6
0.4
0.2
0.0
0.0
0.2
0.4
0.6
0.8
1.0
2
3
4
5
6
7
8
9
10

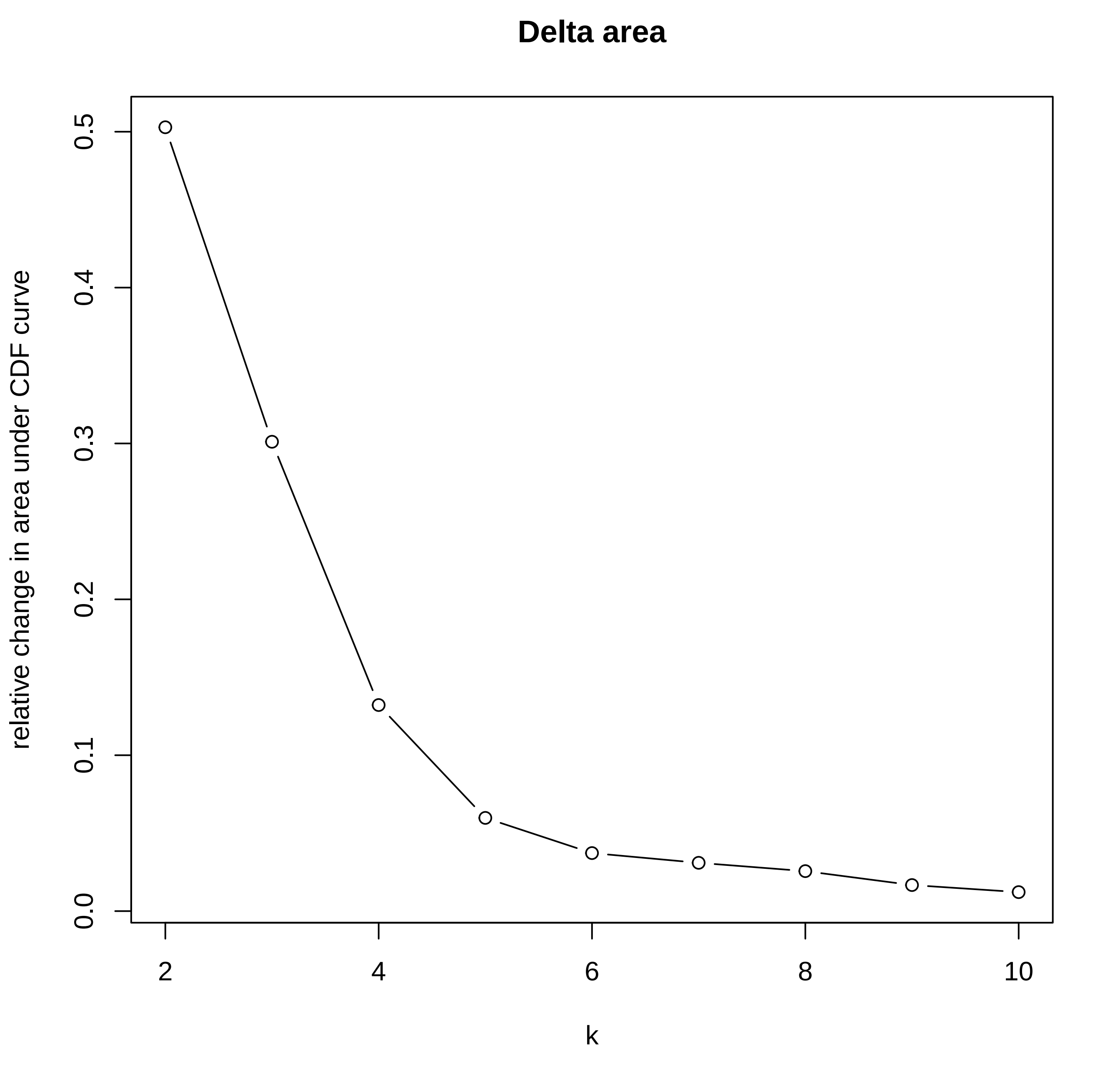
Delta area
relative change in area under CDF curve
0.0
0.1
0.2
0.3
0.4
0.5
2
4
6
8
10
k

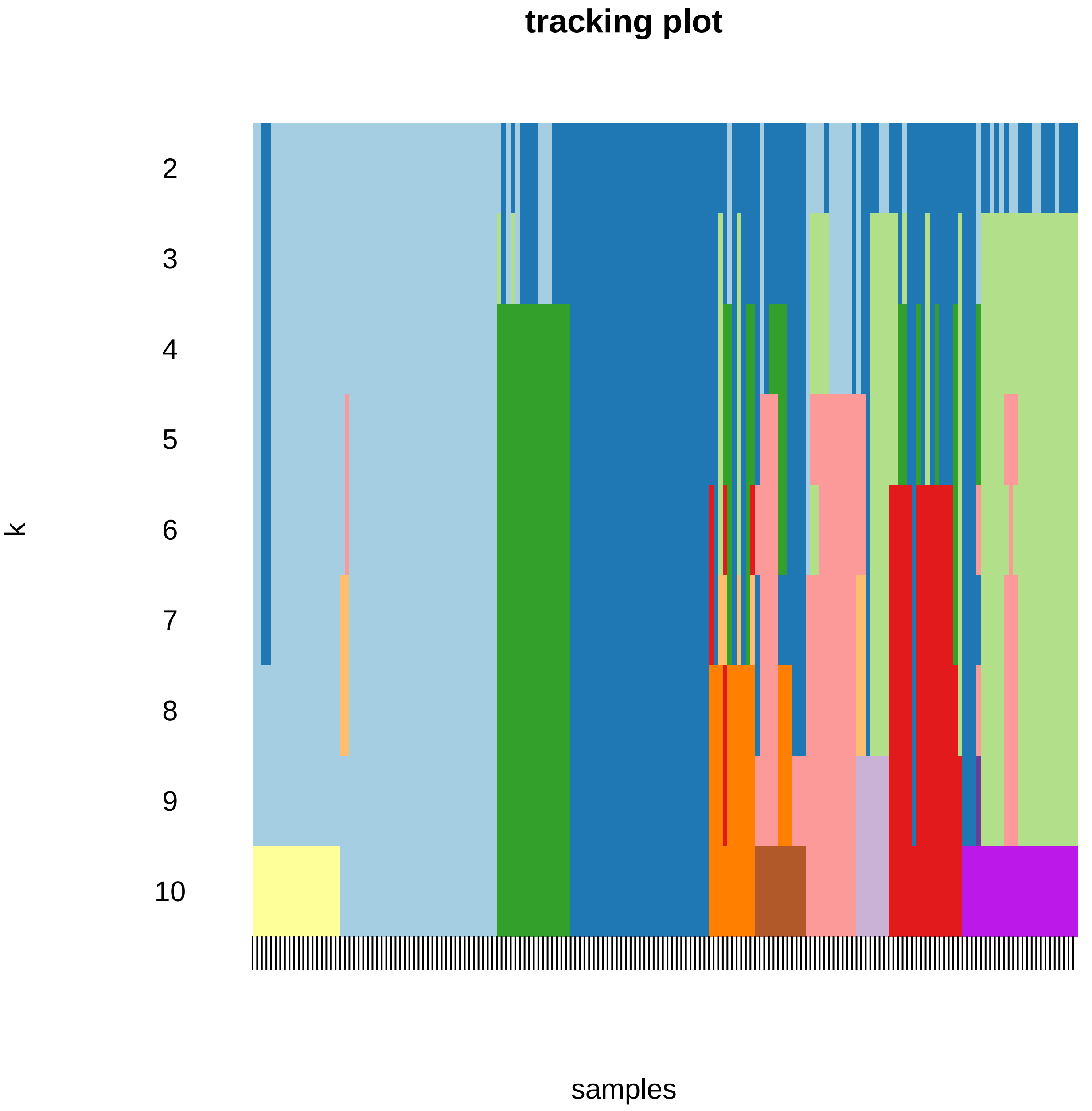
tracking plot
k
2
3
4
5
6
7
8
9
10
samples
