# Supplementary figures and images for: Exploring IRGs as a Biomarker of Pulmonary Hypertension Using Multiple Machine Learning Algorithms
Source: Diagnostics (Basel). 2024 Oct 28;14(21):2398. doi: 10.3390/diagnostics14212398 (PMC11545203; doi:10.3390/diagnostics14212398)

## Sample clustering to detect outliers

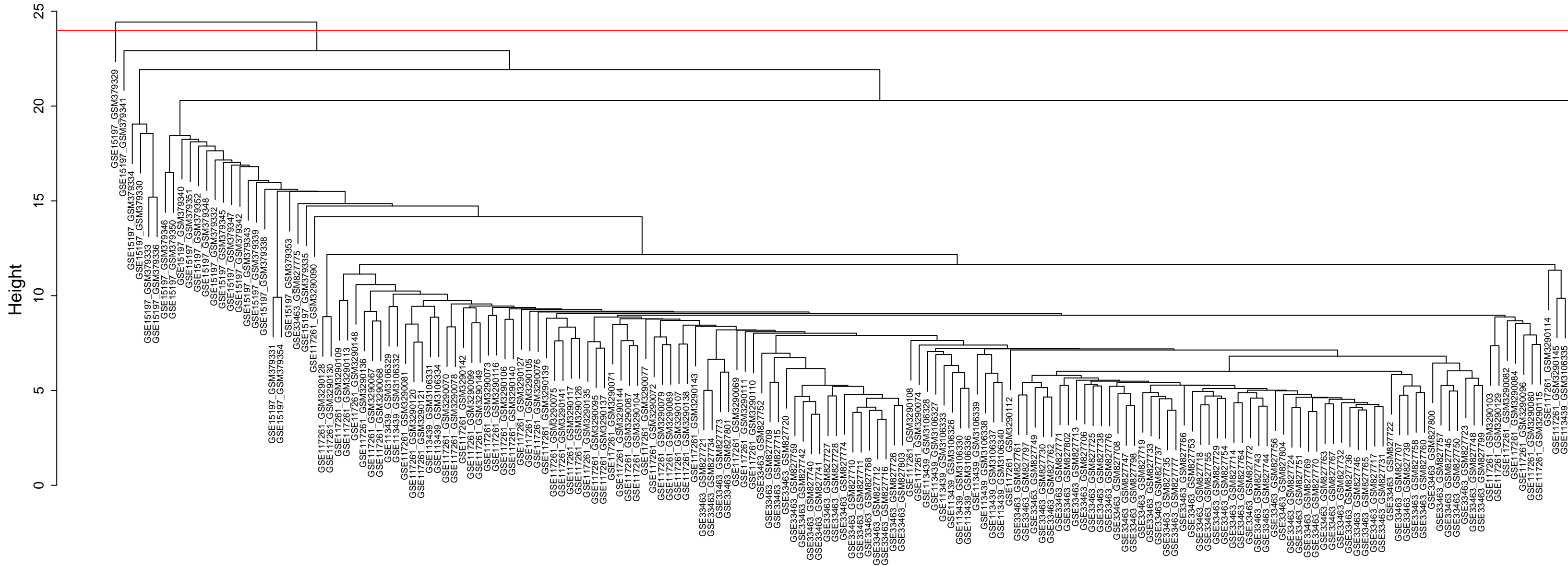

Supplement: Supplementary file 1 [file diagnostics-14-02398-s001.zip › Figure S2.pdf]

**nFeature\_RNA**

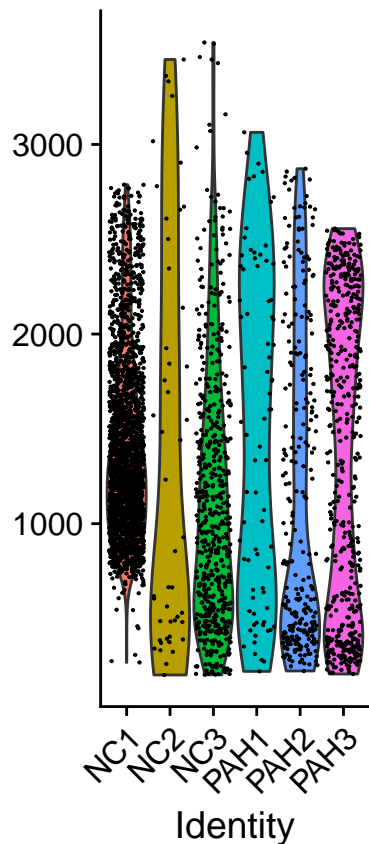

**nCount\_RNA**

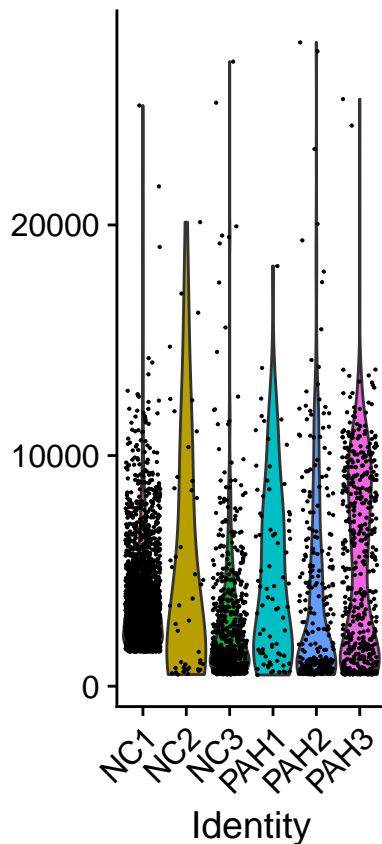

**percent.mt**

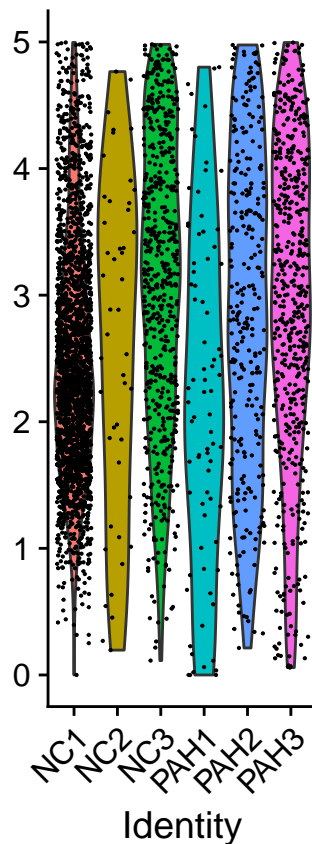

**percent.rb**

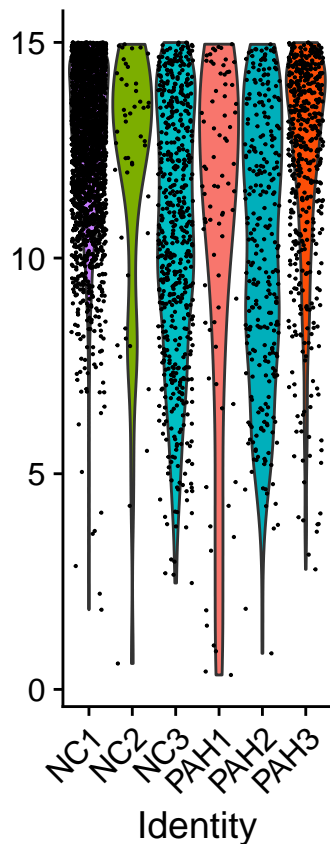

Supplement: Supplementary file 1 [file diagnostics-14-02398-s001.zip › Figure S3.pdf]

**-0.1**

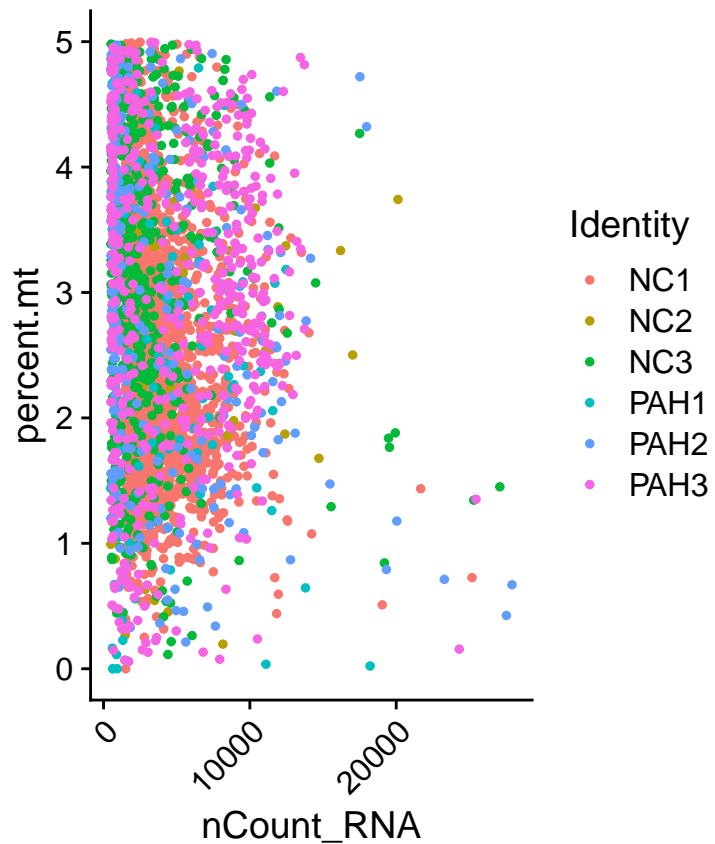

**0.17**

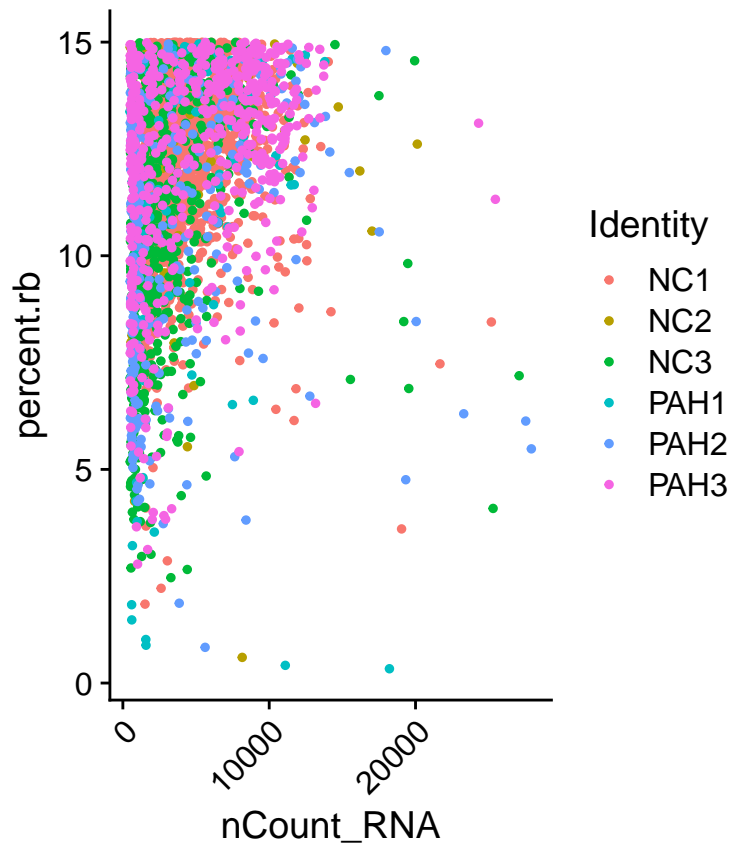

**0.85**

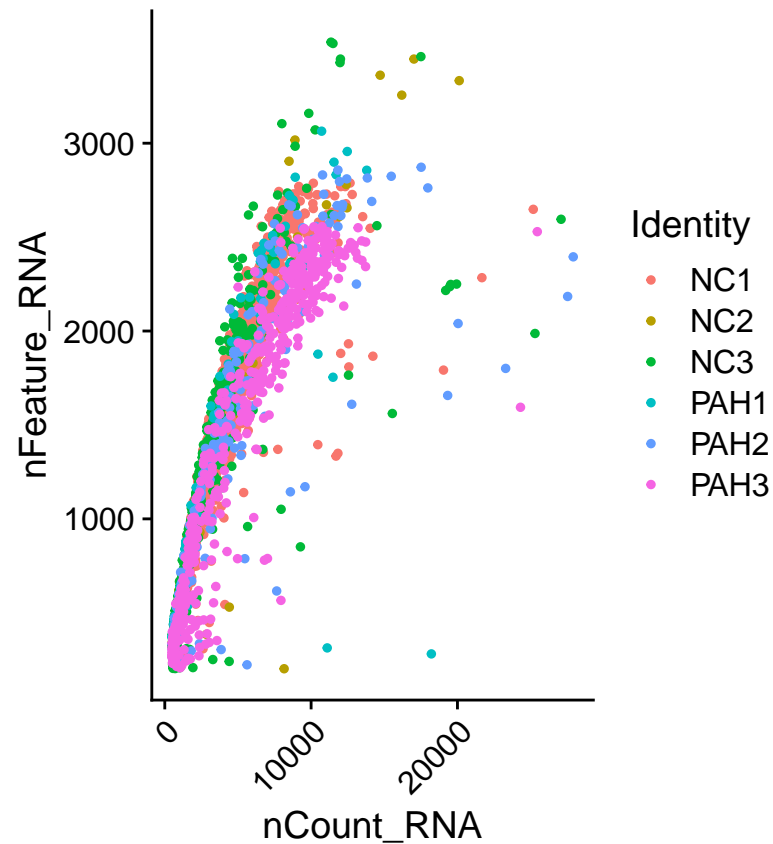

Supplement: Supplementary file 1 [file diagnostics-14-02398-s001.zip › Figure S4.pdf]

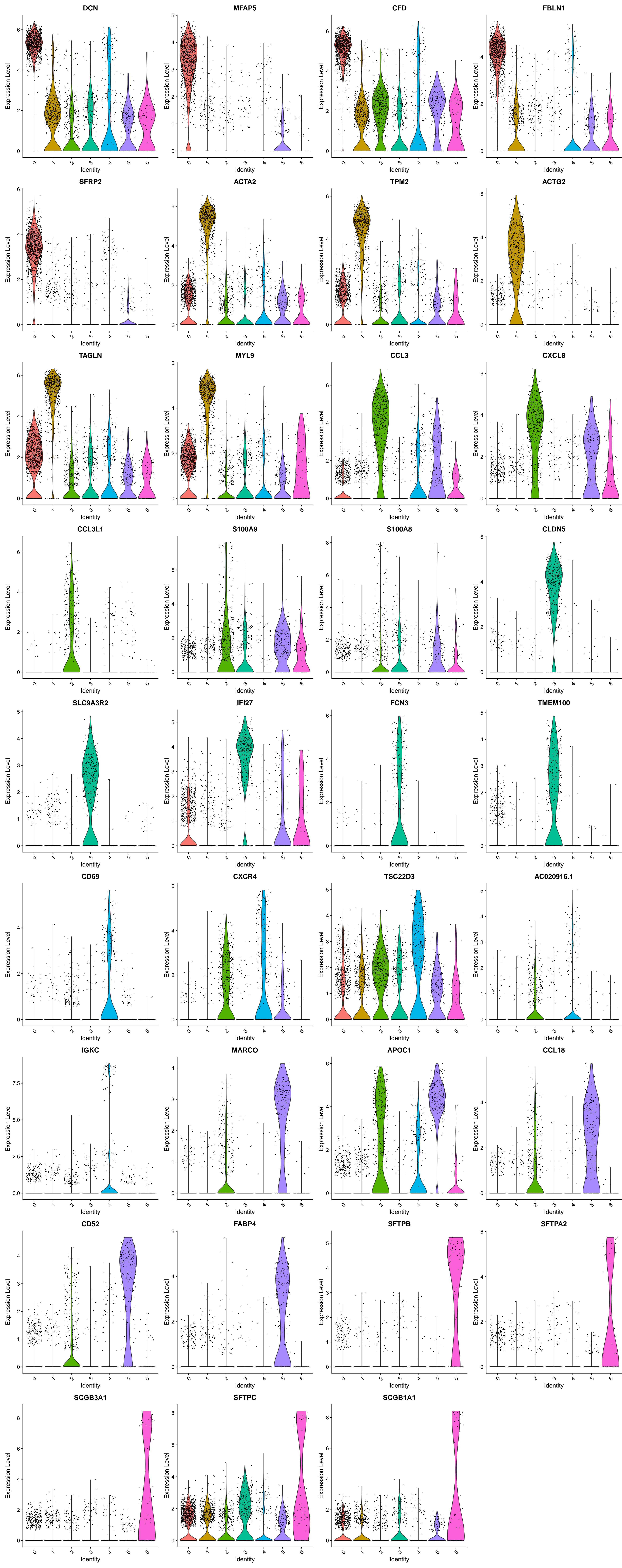

Supplement: Supplementary file 1 [file diagnostics-14-02398-s001.zip › Figure S5.pdf]

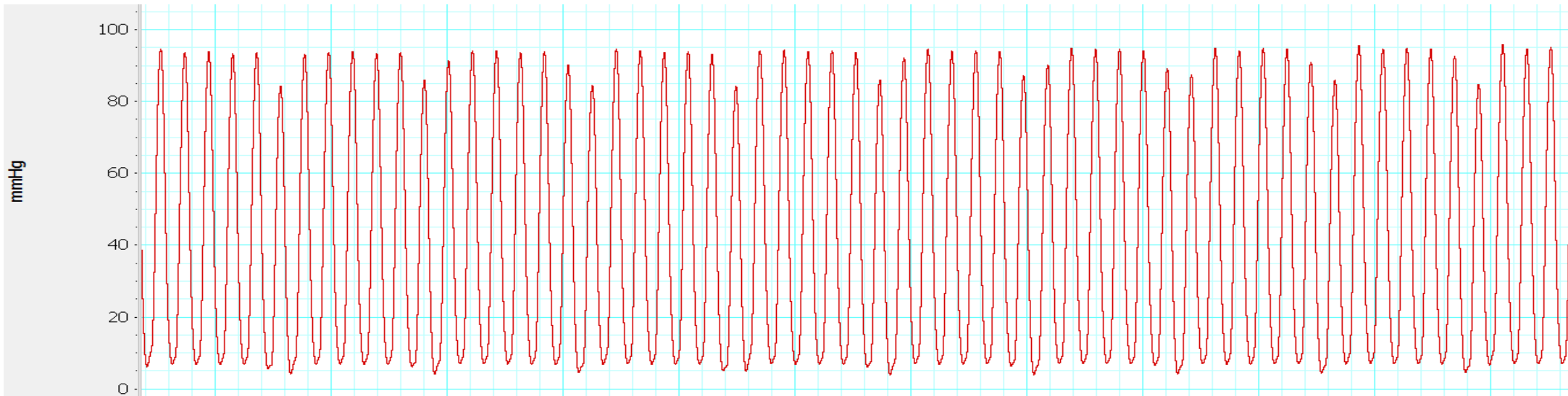

PAH

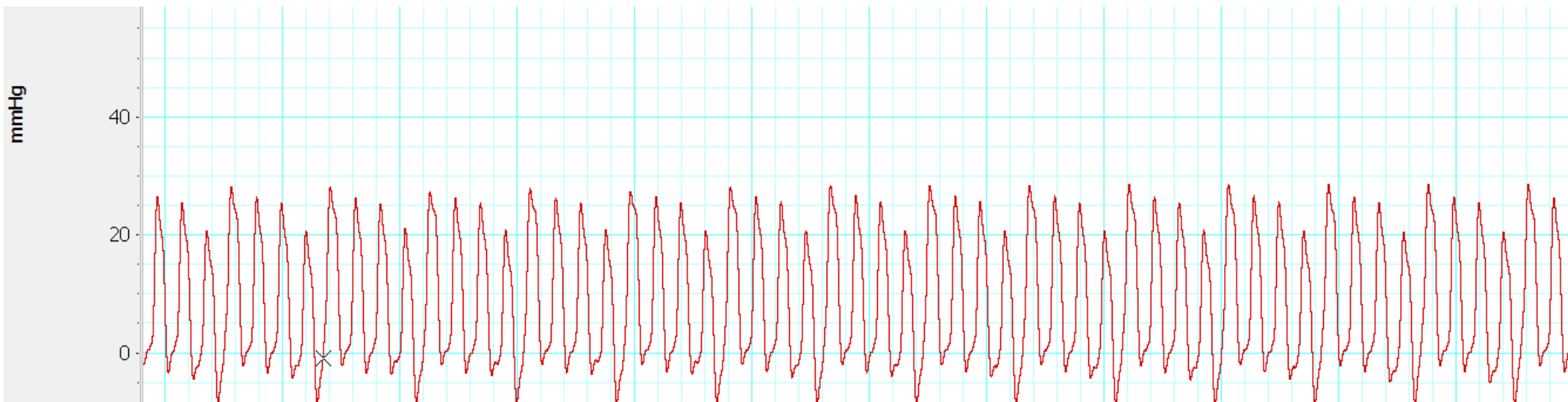

NC

Supplement: Supplementary file 1 [file diagnostics-14-02398-s001.zip › Figure S6.pdf]
